# Effective transport network driven by tortuosity gradient enables high-electrochem-active solid-state batteries

**DOI:** 10.1093/nsr/nwac272

**Published:** 2022-11-28

**Authors:** Qing-Song Liu, Han-Wen An, Xu-Feng Wang, Fan-Peng Kong, Ye-Cai Sun, Yu-Xin Gong, Shuai-Feng Lou, Yi-Fan Shi, Nan Sun, Biao Deng, Jian Wang, Jia-Jun Wang

**Affiliations:** Ministry of Industry and Information Technology (MIIT) Key Laboratory of Critical Materials Technology for New Energy Conversion and Storage, School of Chemistry and Chemical Engineering, Harbin Institute of Technology (HIT), Harbin 150001, China; Chongqing Research Institute of HIT, Chongqing 401135, China; Ministry of Industry and Information Technology (MIIT) Key Laboratory of Critical Materials Technology for New Energy Conversion and Storage, School of Chemistry and Chemical Engineering, Harbin Institute of Technology (HIT), Harbin 150001, China; Ministry of Industry and Information Technology (MIIT) Key Laboratory of Critical Materials Technology for New Energy Conversion and Storage, School of Chemistry and Chemical Engineering, Harbin Institute of Technology (HIT), Harbin 150001, China; Ministry of Industry and Information Technology (MIIT) Key Laboratory of Critical Materials Technology for New Energy Conversion and Storage, School of Chemistry and Chemical Engineering, Harbin Institute of Technology (HIT), Harbin 150001, China; Ministry of Industry and Information Technology (MIIT) Key Laboratory of Critical Materials Technology for New Energy Conversion and Storage, School of Chemistry and Chemical Engineering, Harbin Institute of Technology (HIT), Harbin 150001, China; Ministry of Industry and Information Technology (MIIT) Key Laboratory of Critical Materials Technology for New Energy Conversion and Storage, School of Chemistry and Chemical Engineering, Harbin Institute of Technology (HIT), Harbin 150001, China; Ministry of Industry and Information Technology (MIIT) Key Laboratory of Critical Materials Technology for New Energy Conversion and Storage, School of Chemistry and Chemical Engineering, Harbin Institute of Technology (HIT), Harbin 150001, China; Ministry of Industry and Information Technology (MIIT) Key Laboratory of Critical Materials Technology for New Energy Conversion and Storage, School of Chemistry and Chemical Engineering, Harbin Institute of Technology (HIT), Harbin 150001, China; Ministry of Industry and Information Technology (MIIT) Key Laboratory of Critical Materials Technology for New Energy Conversion and Storage, School of Chemistry and Chemical Engineering, Harbin Institute of Technology (HIT), Harbin 150001, China; Shanghai Institute of Applied Physics, Chinese Academy of Sciences, Shanghai 201204, China; Canadian Light Source Inc., University of Saskatchewan, Saskatoon, SK S7N 2V3, Canada; Ministry of Industry and Information Technology (MIIT) Key Laboratory of Critical Materials Technology for New Energy Conversion and Storage, School of Chemistry and Chemical Engineering, Harbin Institute of Technology (HIT), Harbin 150001, China; Chongqing Research Institute of HIT, Chongqing 401135, China

**Keywords:** all-solid-state batteries, thick electrodes, lithiation/delithiation heterogeneity, tortuosity gradient, synchrotron techniques

## Abstract

Simultaneously achieving high electrochemical activity and high loading for solid-state batteries has been hindered by slow ion transport within solid electrodes, in particular with an increase in electrode thickness. Ion transport governed by ‘point-to-point’ diffusion inside a solid-state electrode is challenging, but still remains elusive. Herein, synchronized electrochemical analysis using X-ray tomography and ptychography reveals new insights into the nature of slow ion transport in solid-state electrodes. Thickness-dependent delithiation kinetics are spatially probed to identify that low-delithiation kinetics originate from the high tortuous and slow longitudinal transport pathways. By fabricating a tortuosity-gradient electrode to create an effective ion-percolation network, the tortuosity-gradient electrode architecture promotes fast charge transport, migrates the heterogeneous solid-state reaction, enhances electrochemical activity and extends cycle life in thick solid-state electrodes. These findings establish effective transport pathways as key design principles for realizing the promise of solid-state high-loading cathodes.

## Teaser

A tortuosity-gradient thick electrode was developed to promote fast ion transport and mitigate the heterogeneous solid-state reaction, leading to excellent electrochemical performance for solid-state batteries.

## INTRODUCTION

Tremendous interest in high-security and high-energy batteries in energy-storage systems has led to the rapid development of all-solid-state lithium batteries (ASSLBs) [1–3]. Despite great advances in the exploration of new solid-state electrolytes, the electrochemical performance of solid-state batteries is still inferior to that of commercial lithium-ion batteries (LIBs) with liquid electrolytes owing to high ion-transport resistance over solid-state electrode–electrolyte interfaces, in particular those employing high-loading cathodes to achieve high energy density [4,5]. Fast solid-state ion transport in high-loading electrodes remains a vital challenge for ASSLBs [6–8].

In a conventional solid-state electrode fabrication in which active materials are mixed with inactive components (conductive carbon, solid-state electrolyte binder) and slurry coated on current collectors, with an increase in electrode thickness, some critical challenges remain unsolved, including: (i) the proportionally increased electronic–ionic transport distance and resistance, (ii) convoluted transport pathways, (iii) severe physical-contact loss and (iv) the low utilization of active materials [9–13]. Unlike liquid electrolytes in traditional liquid-electrolyte batteries that can easily be infiltrated through the interface and porous electrodes, lithium ions can only transport slowly across the solid–solid interfaces in ASSLBs [14–16]. When the electrode thickness increases, the slow ion transport significantly increases the diversity of the particle environment, which poses significant hurdles to the charge-transfer kinetics, the active-materials utilization and the long cycling stability for solid-state batteries [17,18]. In a solid-state battery, the ionic transport kinetics within each particle are governed by local electrochemical potentials, which show a strong dependence on the relative positioning of the individual particle within the electrode architecture [19,20]. As the electrode thickness increases, the long transport distance leads to an ion-concentration gradient along the longitudinal axis and induces heterogeneous electrochemical reactions, deteriorating battery electrochemical performance [21,22]. The influence essentials of the ion-concentration gradient at thick solid electrodes and the underlying mechanism are still ambiguous.

Advanced characterization and analysis methods demonstrate that depth-dependent microstructural engineering effort could be effective in mitigating the degradation of thick cathode electrodes [23,24]. Electrode-structure designs with low porosity, particle morphology or additives have shown improved performance for ASSLBs [25–27]. However, solid–solid ion-transport kinetics are mainly limited by physical interfacial contacts, ionic conductivity and ion-transport paths [28]. When the electrode thickness increases, the coupling of ion-transport and reaction kinetics should be considered as a criterion for designing the electrode structure.

In this work, we provide 3D-visualized insights into the nature of ion transport in solid-state batteries. Enabled by a combination of synchrotron X-ray ptychography, X-ray tomography and machine-learning analysis, we revealed a thickness-dependent solid-state ion-transport behavior that is related to the high tortuous longitudinal transport pathways in the ASSLBs. The high tortuous transport pathways induce the apparent discrepancy between the slow lithium-ion transport and lithiation/delithiation, leading to excessive/incomplete lithiation/delithiation of local particles and performance failure of solid electrodes. To address the sluggish charge kinetics that accompany the increase in electrode thickness, we fabricate a tortuosity-gradient hierarchical electrode (TGH-electrode) composed of a fast transport layer (FTL) with an ionic percolation network and reaction equilibrium layer (REL) with large-sized particles. The full cells with TGH-electrodes exhibit highly improved specific capacity (15%) and cycle life (100 cycles) under a thick solid-state cathode (100-μm thickness, 20 mg cm^−2^).

## RESULTS

### Deteriorated electrochemical performance at thick solid electrode

**Figure 1.**
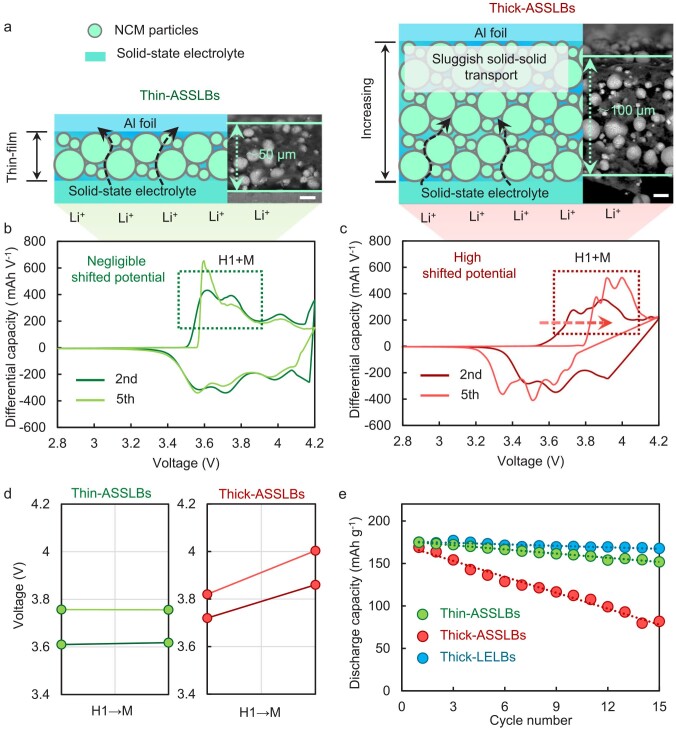
Poor electrochemical properties of thickened solid-state batteries. (a) Schematic diagram of thin-ASSLBs and thick-ASSLB. (b and c) dQ/dV profiles at the second cycle and fifth cycle for (b) thin-ASSLBs and (c) thick-ASSLBs. (d) The potential of H1 to M at the second cycle and fifth cycle in thin-ASSLBs and thick-ASSLBs. (e) Comparison of the cycling stability of thin-ASSLBs, thick-ASSLBs, and thick-LELBs under 0.1C. The loadings of thin-ASSLBs, thick-ASSLBs, and thick-LELBs are ≈10, 20, and 20 mg cm^−2^, respectively.

The electrodes constructed from commercial NCM811 polycrystalline particles were selected as the model electrode to study the lithium-ion transport inside the solid-state electrode. The average size of polycrystalline particles is ∼10 μm and the XRD result is shown in Supplementary Fig. S1. Spherical secondary particles are composed of randomly oriented primary particles, as shown in Supplementary Fig. S2. After homogenously mixing NCM811 materials with conductive agents and binder (polyethylene oxide (PEO)/lithium bis(trifluoromethane sulfonimide) (LiTFSI)), the slurry was cast on clean Al foil to obtain thin and thick electrodes (Fig. 1a). The electrode particles are uniformly embedded in the solid-state electrolytes, which can be used to construct the continuous solid–solid contact network (Supplementary Fig. S3). Flexible solid polymer electrolyte films consisting of PEO and LiTFSI were prepared by using the solution-pouring method (Supplementary Fig. S4) [29]. The thickness of the solid electrolyte was 107 μm (Supplementary Fig. S5) and the ionic conductivity was 2.54 × 10^−4^ S cm^−1^ (Supplementary Fig. S6).

We first utilized a differential capacity curve (dQ/dV) to investigate the lithiation/delithiation properties of solid-state batteries. The dQ/dV profiles of thin all-solid-state lithium batteries (thin-ASSLBs) exhibit a negligible shifted potential range before and after cycling as shown in Fig. 1b. On the contrary, the cycled electrode potential shifts significantly as the thickness of the electrode increases (Fig. 1c). The delithiation process proceeds in the region where the potential was raised by 0.11 V in cycled thick solid-state batteries (thick-ASSLBs) (Fig. 1c and d). Consequently, thick-ASSLBs also showed a lower capacity retention of ≈40% than thin-ASSLBs (≈100%) after 15 cycles (Fig. 1e). In sharp contrast, there is no obvious potential shift and capacity decline in thick liquid-electrolyte lithium batteries (thick-LELBs) (Supplementary Figs S7b, S7c and S8, and Fig. 1e).

Differently from the full infiltration of liquid electrolyte in electrodes, solid-state electrodes lack synchronized lithium-transport channels and therefore lithium transport is sluggish across the solid–solid interface inside ASSLBs [30,31]. When the longitudinal distance of the solid-state electrode increases, due to the slow and consistent ionic transport processes and long transport paths, lithium ions cannot transport smoothly between a solid-state electrolyte and a current collector, resulting in the formation of a lithium-ion-concentration gradient along the longitudinal axis of the thick electrode in ASSLBs. Therefore, the thick electrodes in solid-state batteries present capacity decay behavior that is distinctive from liquid batteries. The dramatic potential buildup leads to a continuously increased voltage environment at the cathode side over repetitive cycles, causing serious capacity decay in thick-ASSLBs.

### Ionic transport barriers in thick-ASSLBs

**Figure 2.**
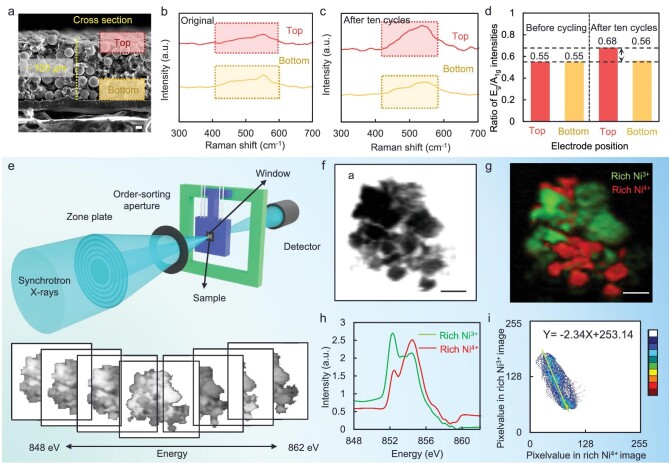
Ionic transport barriers in thick-ASSLBs. (a) SEM image of solid-state electrode. The scale bar represents 10 μm. (b and c) Raman spectra of the NCM particles at the top and bottom of (b) original and (c) cycled thick-ASSLBs. (d) Ratio of E_g_/A_1 g_ intensities at the top and bottom of original (left) and cycled (right) thick-ASSLBs. (e) Schematic of synchrotron STXM. (f) STXM morphology image of the cycled regular-ASSLBs. The scale bar represents 2 μm. (g) PCA analysis for different components of cycled ASSLBs (the scale bar represents 2 μm) and (h) the corresponding average XAS spectra of the Ni L3-edge. (i) Statistical correlation between rich Ni^3+^ and rich Ni^4+^ in Fig. 2g.

To identify the potential buildup related to ionic transport in thick-ASSLBs, we performed Raman spectroscopy to monitor the evolution of the electrode. The A_1 g_ mode and E_g_ mode of Raman spectroscopy can characterize local structural transitions such as the transition from the monoclinic to the hexagonal phase [32,33]. Here, the original and cycled electrode were both divided into top and bottom regions according to the distance from the current collector and the respective spectra were acquired from the marked area (Fig. 2a) to understand the delithiation behaviors along the longitudinal direction of the thick-ASSLBs. The shapes of the spectra before cycling were similar at the top and the bottom (Fig. 2b), although their Raman spectra changed significantly (Fig. 2c) after 10 cycles. The intensity of the A_1 g_ peak was considerably higher than the intensity of the E_g_ peak in the bottom area, but the boundary between A_1 g_ and E_g_ became blurred in the top area. In addition to a considerable change in the shape of the Raman spectrum, it is worth noting that the delithiation heterogeneity was clearly exhibited by the intensity ratio of the E_g_/A_1 g_ of the thick electrode (Fig. 2d). The value at the top of the cycled thick electrode was higher than at the bottom, which demonstrates that the amounts of delithiation from the electrode top area were larger than the amount of delithiation inside the electrode. The longitudinal delithiation heterogeneity of the cathode could induce the top electrode potential of thick cathodes to reach the charge high voltage earlier than the bottom. The relatively high potential environment at the top of the electrode could cause morphological collapse and structural deterioration of the electrode.

The electrochemical stability in the ASSLBs is related to the chemical state and element distribution of the solid-state electrode [34]. Therefore, we applied scanning transmission X-ray microscopy (STXM) to reveal the local Ni-element distribution of the degraded cathode in the ASSLBs (Fig. 2e). The morphology image in Fig. 2f exhibits a secondary particle consisting of primary particles and microcracks are generated after cycling. The principal component analysis (PCA) mapped out the distribution of the chemical phase and distinguished between rich Ni^3+^ areas (green) and rich Ni^4+^ areas (red) in Fig. 2g. The corresponding average X-ray absorption spectra (XAS) spectra at the Ni L-edge are displayed in Fig. 2h. The results show that the chemical state of the Ni on the secondary particles is non-uniform and the rich Ni^3+^ and Ni^4+^ regions have obvious boundaries. Meanwhile, the correlation between the rich Ni^3+^ and the rich Ni^4+^ is negative (Fig. 2i), which suggests that Ni^3+^ changes to Ni^4+^ during the charging process. A more negative correlation in the particles indicates a lower amount of Li extraction and higher delithiation heterogeneity. Therefore, the delithiation heterogeneity occurs even on a single secondary particle in the thick-ASSLBs due to the slow and uniform ion-transport kinetics.

*In situ* electrochemical impedance spectroscopy (EIS) was applied in different states of charge (Supplementary Fig. S9a and b) to evaluate the Li-transport kinetics in the thick-ASSLBs. As shown in Supplementary Fig. S9b, the EIS results show that the total resistance decreases first and then increases in the initial charging of thick-ASSLBs [35]. In contrast, EIS results of thin-ASSLBs and thick-LELBs only show a negligible increase in Supplementary Fig. S10a and b. In the thick-ASSLBs, the cathode-interface resistance decreases significantly at the beginning of the charge, which can be attributed to the activation of the pristine solid interface [36]. The subsequent increase in interface resistance is the result of the thickening of the solid electrolyte interface (SEI) due to the relatively high-voltage environment near the top of the electrode. The continuous thickening of the SEI layer will form a surface resistance layer, which hinders the diffusion of lithium ions, resulting in a rise in the impedance on the electrode side [37].

The short transport distance at the top enables the particles to achieve a deep delithiation level and the long transport distance and slow transport rates at the bottom of thick electrode make the delithiation of the particles slower. In general, as the thickness of the electrode increases, the slow and uniform ion transport in the electrode causes a delithiation gradient on the longitudinal axis of the electrode. The delithiation heterogeneity can form a Li^+^-concentration gradient along the longitudinal axis in the electrode, which ultimately leads to battery failure (Supplementary Fig. S11).

### Improved performance with the TGH-electrode

**Figure 3.**
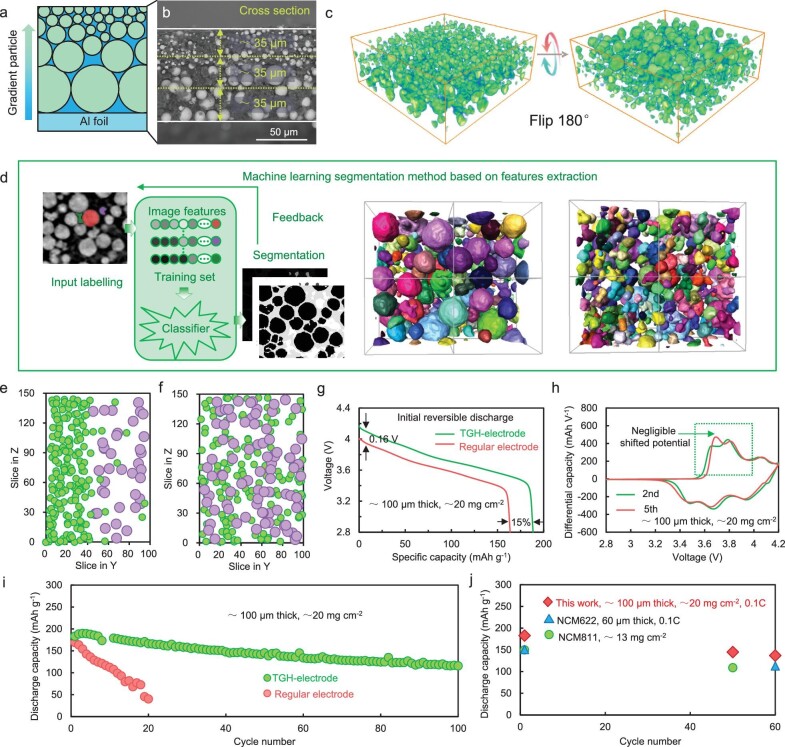
Improved electrochemical performances and 3D tomography analysis of TGH-electrode. (a) Schematic illustration of the TGH-electrode. (b) SEM image of the TGH-electrode. (c) Synchrotron X-ray tomography reconstruction with volume rendering shows the 3D microstructure of the TGH-electrode. (d) Schematic illustration of the machine-learning model for particle identification and segmentation. Spatial distribution of the LiNi_0.8_Co_0.1_Mn_0.1_O_2_ (NMC) particles of (e) TGH-electrode and (f) regular cathodes in the direction normal to the current collector. Green circles: >12-μm particles; purple circles: <12-μm particles. (g) Initial reversible discharge curves of the TGH-electrode at 0.1C. (h) dQ/dV profiles at the second cycle and fifth cycle for the TGH-electrode. (i) Cycling stability comparison between the TGH-electrode and regular cathodes under 0.1C. (j) Cycling performance and specific capacity comparison with relevant literature.

In thick-ASSLBs, lithium-ion transport is slow along the longitudinal direction of the electrode, resulting in potential buildup on the electrode surface close to the solid-state electrolyte. The non-uniform potential distribution leads to delithiation heterogeneity in the electrodes, which leads to the unrecoverable loss of capacity in thick-ASSLBs. Hence, to overcome these issues, we propose a TGH-electrode with the coupling of low and high tortuosity to recast Li^+^-transport pathways and balance the rates of lithium transport and consumption (Fig. 3a). We first sieved commercial LiNi_0.8_Co_0.1_Mn_0.1_O_2_ (NCM) materials into large particles (15–20 μm), medium particles (8–15 μm) and small particles (<8 μm). Then, large particles were coated on the side close to the current collector as the REL, medium particles and small particles were sequentially coated on the REL as the FTL. The NCM particles were recast into the TGH-electrode in a tortuosity-gradient arrangement in the electrode (Fig. 3b).

A deep understanding of the role that the TGH-electrodes play in ASSLBs requires advanced characterization methods. Therefore, we utilize synchrotron X-ray imaging technology with a large field of view to analyse the microstructure of the TGH-electrode [38]. As shown in Fig. 3c, the 3D rendering of the NCM particles displays different viewing angles in the TGH-electrode, where the REL and FTL are observed, which is consistent with the scanning electron microscopy (SEM) images (Fig. 3b). However, due to the complexity of the composite electrode, it is difficult to distinguish small particles in the electrode using the traditional gray-scale value-based segmentation method (Supplementary Fig. S12). The morphological discrimination of the different phases using a machine-learning segmentation method (Fig. 3d) within the TGH-electrode allows complete image analysis to investigate the electrode microstructures [39,40]. Hence we can visualize the distribution of the active-material particles in the longitudinal direction of the current collector (Fig. 3e and f) and the results indicate that the TGH-electrode presents a TGH structure, small particles for the FTL and large particles for the REL.

To determine the feasibility of the TGH-electrode strategy, we conducted electrochemical measurements. The discharge capacity of the TGH-electrode (≈188 mA g^−1^) is 15% higher than that of regular cathodes (≈163 mA g^−1^) after activation (Fig. 3g) and the polarization voltage is 0.16 V lower than that of regular cathodes, which demonstrates that the TGH structure can effectively improve the ion-transport and delithiation kinetics of the electrode. A dQ/dV test was used to study the potential accumulation phenomenon. Before and after the cycle, the potential showed a negligible potential shift (Fig. 3h), which indicated that there was uniform potential distribution in the TGH-electrode. In terms of cycle stability, the capacity of regular cathodes is only 40 mAh g^−1^ after 20 cycles. In addition, the small-particle electrode shows the highest initial capacity (197 mAh g^−1^) (Supplementary Fig. S13), which demonstrates the fastest ionic transport. However, the capacities are also only 28 mAh g^−1^ after 100 cycles, which is because of the delithiation heterogeneity caused by consistent ionic transport processes inside the electrode, even for small-particle electrodes. In contrast, the capacity of the TGH-electrode is still 116 mAh g^−1^ under ≈20 mg cm^−2^ of loading after 100 cycles, which indicates that the deterioration in the thick electrodes is suppressed well (Fig. 3i and Supplementary Fig. S14). The subsequent Raman spectra also showed that the spectra of the cycled TGH-electrode were consistent at the top and the bottom areas (Supplementary Fig. S15a and b), which indicates that there is no delithiation heterogeneity in the TGH-electrode. The electrochemical performances of the TGH-electrode also have advantages compared with the polymer solid-state batteries that have been reported (Fig. 3j) [41,42].

### The ion-transport mechanism of the TGH-electrode

**Figure 4.**
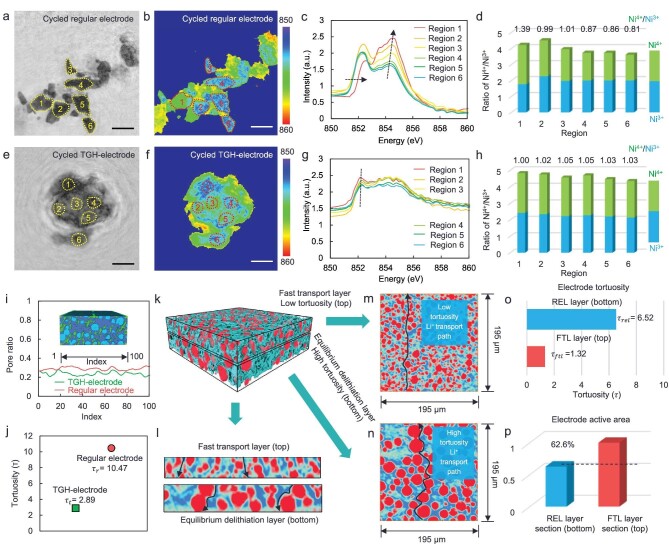
The ion-transport equilibrium of the TGH-electrode. (a) Ptychography STXM amplitude (optical density) image and (b) elemental distribution mapping at the Ni edges of cycled regular cathodes. The scale bars represent 2 μm. (c) XAS spectra of the Ni L3-edge and (d) quantification of the Ni^3+^ and Ni^4+^ in different regions extracted from Fig. 4c. (e) Ptychography STXM amplitude (optical density) image and (f) elemental distribution mapping at the Ni edges of the cycled TGH-electrode. The scale bars represent 2 μm. (g) XAS spectra of the Ni L3-edge and (h) quantification of the Ni^3+^ and Ni^4+^ in different regions extracted from Fig. 4g. (i) The pore distribution of regular electrode and TGH-electrode. (j) The tortuosity of regular electrode and TGH-electrode. (k) Synchrotron X-ray tomography reconstruction with volume rendering shows that the TGH-electrode is composed of FTL and REL. (l) The 2D central longitudinal virtual slice of the FTL and REL. The 2D transverse slice of (m) the FTL and (n) the REL. (o) The tortuosity of the FTL and REL. (p) The active area of the FTL and REL.

Soft X-ray ptychography chemical imaging can overcome the limit of optics-induced image-forming spatial resolution to reach the Raleigh limit spatial resolution (Supplementary Fig. S16) [43,44]. Therefore, the ptychography technique is a unique technology for high-resolution chemical and electronic structure characterization of battery materials. In the cycled regular cathodes, the ptychography results show that the secondary particles are fragmented (Fig. 4a), which is due to the collapse of the particle structure induced by excessive voltage accumulation. The PCA mapped out the chemical-phase distribution and showed the Ni chemical state heterogeneity in the selected Regions 1–6 (Fig. 4b). The Ni L-edge XAS (Fig. 4c) are also extracted from the corresponding areas for fine structure analysis. Compared with other regions, the Ni L-edge spectrum of Region 1 exhibits a ∼0.4-eV edge energy shift toward higher energy, which reflects an oxidation state increase for Ni in Region 1. In addition, Region 1 also reveals a higher peak intensity ratio of ∼1.39 of Ni^4+^/Ni^3+^ (Fig. 4d), which indicates that Region 1 has a deeper delithiation level than other areas. The spectra of Regions 3–6 show similar Ni^3+^ peak intensity, but the Ni^4+^ peak intensity gradually decreases and the peak intensity ratio of Ni^4+^/Ni^3+^ is 1.01, 0.87, 0.86 and 0.81, respectively. These results indicate that there is serious delithiation heterogeneity in regular cathodes. In contrast, the result of ptychography chemical imaging demonstrates the entire secondary-particle morphology (Fig. 4e) and uniform Ni chemical state distribution (Fig. 4f) in the TGH-electrode. There is no shift in Regions 1–6 of the Ni L-edge XAS spectrum (Fig. 4g) and the peak intensity ratio of Ni^4+^/Ni^3+^ is similar at ∼1.02 (Fig. 4h). It proves that the TGH strategy is beneficial to the uniformity of delithiation in the TGH-electrode.

With the increase in the thickness of the electrode, excessive longitudinal ion transport has been considered as the major obstacle to the high performance of ASSLBs. To further illustrate the effect of the L^+^-transport path on the electrodes, the connectivity network model (CNM) using synchrotron micro-tomography data was applied to investigate the thick electrode [45]. The machine-learning segmentation method was used to distinguish the Li^+^-transport pathways and then establish the ion CNM (Supplementary Fig. S17). The color-coded heterogeneity of the stick model was correlated with the transport distance in the CNM. The network model shows that the regular cathodes have uniform and long (red color) transport distances throughout the electrode (Supplementary Fig. S17a). In contrast, the FTL in the TGH-electrode has formed a lithium-ion-percolation network with short and dense lithium-ion-percolation pathways (green color) (Supplementary Fig. S17b), which ensure that lithium ions can be transported rapidly to reach all of the storage sites. The fast ion-transport network near the electrolyte side can avoid continuous potential buildup and excessive delithiation of the NCM materials, promoting the stable cycle of the batteries.

In the electrode of LIBs, the tortuosity of the transport path can limit the lithium-ion-transport kinetics [46]. As shown in Supplementary Fig. S18, the three typical geometries A, B and C have different tortuosity factor values ${{\rm{\tau }}}_1$< ${{\rm{\tau }}}_2$< ${{\rm{\tau }}}_3$, which indicates an increase in the ion-transport paths [47,48]. Synchrotron 3D imaging can directly capture the true geometry of the porous electrode and accurately calculate the porosity of the electrode, which is an important parameter related to tortuosity. As shown in Fig. 4i, the regular electrode exhibits larger average porosity of 0.29. However, the TGH-electrode shows a dense average porosity of 0.24. A conventional blocking-electrolyte impedance method was used to measure the difference in electrode tortuosity (Supplementary Fig. S19a–c) [49,50]. The average tortuosity of the regular electrode is determined to be ≈10.47, which is much larger than the average tortuosity of TGH-electrodes at ≈2.8 (Fig. 4j). Using the machine-learning method can accurately identify the electrode as a particle-phase, the carbon and binder domai (CBD) network and pore network (Supplementary Fig. S20a–d), so the tortuosity of the electrode can be directly obtained [51]. The tortuosity obtained from the 3D data also supports the difference in the tortuosity as shown in Supplementary Fig. S21. The statistical analysis of the capacity, thickness and tortuosity is shown in Supplementary Fig. S22. The low tortuosity can form an efficient ion-transport network to transport ions rapidly to electrochemically active sites, thereby enhancing the electrochemical activity and cycle life of the battery.

To investigate the equilibrium mechanism between Li transport and delithiation, synchrotron 3D structure analysis is applied to examine the ion-transport behaviors of the FTL and the REL. As shown in Fig. 4k and l, the 3D rendering of the TGH-electrode displays the different views of slices in the electrode, where it can be observed that the FTL is composed of small particles and the REL is composed of large particles. Owing to the lower tortuosity provided by small particles in the FTL, the FTL has a short vertical ion-transport path (Fig. 4m–o), ensuring that Li^+^ can be transported rapidly from the electrolyte side to the current-collector side. The REL layer has a small specific surface area (Fig. 4p) and only a low Li^+^ flux is required to achieve a specific delithiation level, which provides the possibility of equilibrium between Li^+^ transport and delithiation in the TGH-electrode. The synergy between the FTL and the REL maintains the equilibrium between ion transport and delithiation in the TGH-electrode, improving the cathode utilization and promoting the cycle stability of ASSLBs.

## CONCLUSION

In summary, we quantify the electrochemical heterogeneity of solid electrodes using multimode X-ray microscopy to uncover the deteriorated reaction kinetics mechanism in thick solid-state battery electrodes. The slow ion transport leads to the large discrepancy between slow lithium-ion transport and lithiation/delithiation, which eventually leads to the heterogeneous redox reaction. As the cycle progresses, the high tortuosity in thick solid-state battery electrodes leads to heterogeneous lithiation and stress localization. To address this challenge associated with thick electrodes, we propose a tortuosity-gradient electrode architecture composed of the FTL and REL, forming an efficient ion-transport network to mitigate the imbalance of transport and reaction rates in ASSLBs. The ionic percolation network with short transport pathways in the low-tortuosity layer facilitate lithium-ion transport along the electrode longitudinal direction, while the low active surface in the high-tortuosity layer close to the current collector only needs a small Li^+^ flux to achieve deep lithiation/delithiation. Compared with conventional electrode configurations, the TGH-electrode architecture substantially increases the active-material utilization and cycle life of solid-state batteries. Our work paves a new way for engineering the design of high-energy solid-state lithium batteries upon long-term cycling.

## METHODS

### Electrode synthesis and electrochemical measurements

Detailed electrode synthesis methods and electrochemical measurements are provided in the Supporting Information.

### Physical characterization

#### Transmission X-ray microscopy and data analysis (TXM)

The synchrotron tomography images were collected at the beamline BL13W1, Shanghai Synchrotron Radiation Facility (SSRF) at >20 keV, for ∼900 projections over an angular range of 180° with a field of view of 1000 × 1000 μm (with a 2k × 2k CCD camera binning 2 × 2 camera pixels into one output pixel). The raw data obtained are tomographically aligned and then reconstructed [52]. The 3D visualization and data analysis were performed using the commercial software package Avizo (Thermo Fisher Scientific, Waltham, Massachusetts, USA) [53].

#### Machine-learning-based identification and segmentation

The identification and segmentation of TXM imaging were performed by using a machine-learning method with the Weka [54]. This method is based on the information of the microstructure captured from the image (2D) or volume (3D) data to train the machine-learning model to identify and extract image features. The advantage of the model is that it uses random forest algorithms related to classification tasks and can get ideal results without a lot of computing resources. We optimize the model by combining electrode particles and CBD shape information defined in the manual annotation data. The gray value of the pixel, the adjacent domain of the pixel and the morphology of each phase are used as label data for the training step to obtain the best model that minimizes the error. This recognition model slightly increases the constraints in the segmentation and enhances the accuracy of the recognition target.

#### STXM

The STXM experiments were carried out at the beamline 10ID-1 SM, CLS, with a 10 × 10 μm^2^ (100 × 100 points) scanned image size. We used the aXis2000 software (open-source software, McMaster University, Canada) to perform principal component analysis–cluster analysis (PCA–CA) of NCM particles on the energy-stack data sets of the STXM with the angular-distance-measurement mode (cut-off value 0.01) [55]. The natural groups of spectra can be identified by using this method, then the average spectra can be calculated and the thickness map associated with these spectra can be exhibited.

#### STXM-ptychography

The STXM-ptychography experiments were also carried out at the beamline 10ID-1 SM, CLS. STXM-ptychography uses an outer-zone-width zone plate with a 35-nm width to focus the monochromatic soft X-ray beam to obtain a 5-μm beam spot. To block all but the first-order beam, a 50-μm order-sorting aperture was used. In order to have sufficient overlap of the scanned areas, 0.5-μm incremental raster scans (10 × 10 pixels) were used. A Greateyes CCD camera (2048 × 2052 pixels) cooled to –40°C was used to record diffraction data. To analyse the NCM particles using STXM-ptychography at multiple elemental edges in a single ptychography stack scan, spectro-ptychography, the photon energies were set to be Ni L-edge (845, 852.0 and 854.4 eV, energy resolution of ΔE/E ≈ 1 × 10^−4^) [56].

#### SEM and XRD

The SEM was performed on Phenom Pro to characterize the morphology features of as-prepared products. The crystal structures of as-prepared samples were characterized by XRD (Philips, Holland) using Cu Kα.

## Supplementary Material

nwac272_Supplemental_File
