# Correction to: Effective transport network driven by tortuosity gradient enables high-electrochem-active solid-state batteries

**DOI:** 10.1093/nsr/nwad135

**Published:** 2023-05-10

**Authors:** Qing-Song Liu, Han-Wen An, Xu-Feng Wang, Fan-Peng Kong, Ye-Cai Sun, Yu-Xin Gong, Shuai-Feng Lou, Yi-Fan Shi, Nan Sun, Biao Deng, Jian Wang, Jia-Jun Wang

**Affiliations:** Ministry of Industry and Information Technology (MIIT) Key Laboratory of Critical Materials Technology for New Energy Conversion and Storage, School of Chemistry and Chemical Engineering, Harbin Institute of Technology (HIT), Harbin 150001, China; Chongqing Research Institute of HIT, Chongqing 401135, China; Ministry of Industry and Information Technology (MIIT) Key Laboratory of Critical Materials Technology for New Energy Conversion and Storage, School of Chemistry and Chemical Engineering, Harbin Institute of Technology (HIT), Harbin 150001, China; Ministry of Industry and Information Technology (MIIT) Key Laboratory of Critical Materials Technology for New Energy Conversion and Storage, School of Chemistry and Chemical Engineering, Harbin Institute of Technology (HIT), Harbin 150001, China; Ministry of Industry and Information Technology (MIIT) Key Laboratory of Critical Materials Technology for New Energy Conversion and Storage, School of Chemistry and Chemical Engineering, Harbin Institute of Technology (HIT), Harbin 150001, China; Ministry of Industry and Information Technology (MIIT) Key Laboratory of Critical Materials Technology for New Energy Conversion and Storage, School of Chemistry and Chemical Engineering, Harbin Institute of Technology (HIT), Harbin 150001, China; Ministry of Industry and Information Technology (MIIT) Key Laboratory of Critical Materials Technology for New Energy Conversion and Storage, School of Chemistry and Chemical Engineering, Harbin Institute of Technology (HIT), Harbin 150001, China; Ministry of Industry and Information Technology (MIIT) Key Laboratory of Critical Materials Technology for New Energy Conversion and Storage, School of Chemistry and Chemical Engineering, Harbin Institute of Technology (HIT), Harbin 150001, China; Ministry of Industry and Information Technology (MIIT) Key Laboratory of Critical Materials Technology for New Energy Conversion and Storage, School of Chemistry and Chemical Engineering, Harbin Institute of Technology (HIT), Harbin 150001, China; Ministry of Industry and Information Technology (MIIT) Key Laboratory of Critical Materials Technology for New Energy Conversion and Storage, School of Chemistry and Chemical Engineering, Harbin Institute of Technology (HIT), Harbin 150001, China; Shanghai Institute of Applied Physics, Chinese Academy of Sciences, Shanghai 201204, China; Canadian Light Source Inc., University of Saskatchewan, Saskatoon, SK S7N 2V3, Canada; Ministry of Industry and Information Technology (MIIT) Key Laboratory of Critical Materials Technology for New Energy Conversion and Storage, School of Chemistry and Chemical Engineering, Harbin Institute of Technology (HIT), Harbin 150001, China; Chongqing Research Institute of HIT, Chongqing 401135, China

In the Fig. 1 of ‘Effective transport network driven by tortuosity gradient enables high-electrochem-active solid-state batteries’ (*National Science Review*, Volume 10, Issue 3, 2023, nwac272, https://doi.org/10.1093/nsr/nwac272), the data for dQ/dV profiles and the corresponding potential for thin-ASSLBs were incorrectly provided (Fig. 1b and d left). The corrected version of Fig. 1 is presented below.

**Figure 1.**
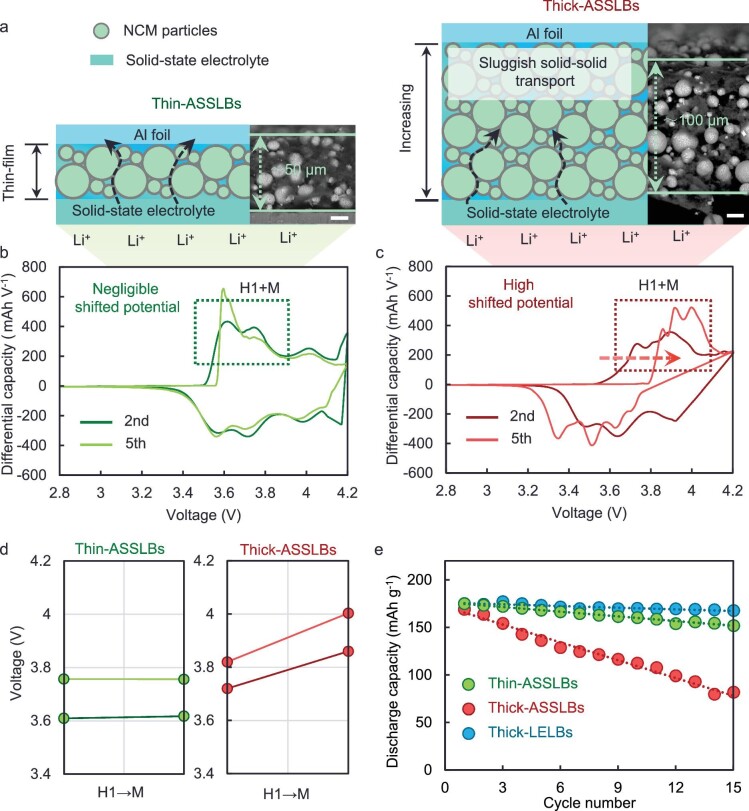
Poor electrochemical properties of thickened solid-state batteries. (a) Schematic diagram of thin-ASSLBs and thick-ASSLB. (b and c) dQ/dV profiles at the second cycle and fifth cycle for (b) thin-ASSLBs and (c) thick-ASSLBs. (d) The potential of H1 to M at the second cycle and fifth cycle in thin-ASSLBs and thick-ASSLBs. (e) Comparison of the cycling stability of thin-ASSLBs, thick-ASSLBs, and thick-LELBs under 0.1C. The loadings of thin-ASSLBs, thick-ASSLBs, and thick-LELBs are ≈ 10, 20, and 20 mg cm^−2^, respectively.

