# Supplementary material for: Effective transport network driven by tortuosity gradient enables high-electrochem-active solid-state batteries
Source: Natl Sci Rev. 2022 Nov 28;10(3):nwac272. doi: 10.1093/nsr/nwac272 (PMC9977374; doi:10.1093/nsr/nwac272)
Supplement: nwac272_Supplemental_File [file nwac272_supplemental_file.docx]

Supporting Information

Effective transport network driven by tortuosity gradient enable high-electrochem-active solid-state batteries

Qingsong Liu, Hanwen An, Xufeng Wang, Fanpeng Kong, Yecai Sun, Yuxin Gong, Shuaifeng Lou, Yifan Shi, Nan Sun, Biao Deng, Jian Wang, Jiajun Wang*

**METHODS**

**Preparation of solid-state electrolyte.** The solid polymer electrolytes were fabricated by a solution casting method. First, Polyethylene oxide (PEO, Mw = 600,000, Aladdin, 98%) and lithium bis(trifluoromethane sulfonimide) (LiTFSI, Aladdin, 99%) were added into anhydrous acetonitrile (ACN) solutions with a molar ratio18: 1 (EO: Li). After fully stirring, the mixture was poured into a polytetrafluoroethylene mold and dried under vacuum at 60 °C for 48 h. Finally, a solid-state electrolyte membrane was obtained through a solution pouring process.

**Preparation of TGH-electrode.** The TGH-electrodes were prepared by a multi-stage coating method. First, the commercial NCM materials were sieved into large particles (15-20 μm) medium particles (8-15 μm), and small particles (less than 8 μm). After that, the three types of particles were mixed with conductivity carbon (Carbon nanotube) and binder (PEO/LiTFSI; Mw= 600,000; EO: Li=18: 1) at a mass ratio of 80:10:10 with ACN, respectively. The large particles slurry was preferentially coated on the Al foil and dried under vacuum at 60 °C for 24 h. Then, the medium and small particles slurries were sequentially coated on the dried electrode foil according to the same method to obtain TGH-electrode.

**Electrochemical measurements.** The cathodes were obtained by mixing commercial LiNi_0.8_Mn_0.1_Co_0.1_O_2_ powder (Zhuhai Coslight Corp.), conductivity carbon (Carbon nanotube) and binder (PEO/LiTFSI; Mw= 600,000; EO: Li=18: 1) at a mass ratio of 80:10:10 with ACN. And the resulting slurry was cast onto Al foil with different thicknesses and dried under vacuum at 60 °C for 24 h. Then, the CR2025 coin-type ASSLBs were assembled in a glovebox filled with Ar where lithium foil as anode, and PEO-based polymer as the solid electrolyte without the addition of any liquid-based electrolyte. The CR2025 coin-type LELBs were assembled using a similar method, except that the solid-state electrolyte was replaced by a polypropylene separator and liquid electrolyte consists of 1 M LiPF_6_ in ethylene carbonate (EC)/dimethyl carbonate (DMC) (1:1 in volume). Galvanostatic cycling measurements were tested on a Neware Battery Cycler (CT-4008T-5V10mA-164, Shenzhen, China) between 2.8- 4.2 V at 60 ℃. The different SOC states of the battery are obtained through the charging capacity as the cut-off condition. Electrochemical Impedance Spectroscopy (EIS) were tested in a CHI660e instrument at the frequency range from 100 kHz to 0.01 Hz.

**The blocking-electrolyte tortuosity test.** The blocking-electrolyte consists of NCM/TGH-electrodes and 20 mM tetrabutylammonium hexafluorophosphate (TBAPF_6_) in ethylene carbonate (EC) and dimethyl carbonate (DMC) (1:1, v/v) as blocking electrolyte.^1,2^ EIS were tested in a CHI660e instrument at the frequency range from 100 kHz to 0.01 Hz. As shown in Figure S13b, the difference between the R_h_ and the R_1_ is ≈ R_ion_/3. The tortuosity calculation formula is as follows:

$$\frac{\tau}{\epsilon}=\frac{R_{ion}\times S\times k_{int}}{l}$$

where $\tau$ is tortuosity factors, $\epsilon$ is the porosity of the electrode (Figure 4i), S is the cross-sectional area of the cell (1.538 cm^-2^), k_int_ is the intrinsic conductivity of the electrolyte (1.74 mS cm^-1^),^1,2^ l is the thickness of the electrode (100 μm).

**The ionic conductivity of solid-electrolyte.** The ionic conductivity of as prepared solid-state electrolyte was tested in an electrochemical working station (CHI660E). The SS|SSE|SS batteries were assembled with stainless steel sheets and as prepared solid-state electrolytes. The ionic conductivity calculation formula is as follows:

$$\sigma=\frac{L}{RS}$$

where $\sigma$ is ionic conductivity of as prepared solid-state electrolyte, the *L* is the thickness (0.0107 cm) of as prepared solid-state electrolyte, *R* is bulk resistance (21Ω), and S is the contact area (2.01 cm^2^) between steel sheet and as prepared solid-state electrolyte.

**The statistical correlation analysis of elements.** The ImageJ/Fiji plugin ScatterJ was used to perform statistical correlation analysis on elements. First, in the rectangular coordinate system, the grayscale values (proportional to OD) of the corresponding pixels of the two-element distribution maps are plotted as horizontal and vertical coordinates. After that, the linear degree and distribution trend of scattered points was used to analyze the overall correlation among the elements in the sample.

**The Raman test.** The Raman data were obtained by a Raman Spectrometer (Via, Renishaw). First, the cycled batteries were disassembled to obtain electrode sheets in an argon-filled glove box. After that, the electrode sheets were cut open with a scalpel to obtain the cross-sections. Finally, in the optical window of the Raman Spectrometer, select the top and bottom of the electrode to perform the Raman test.


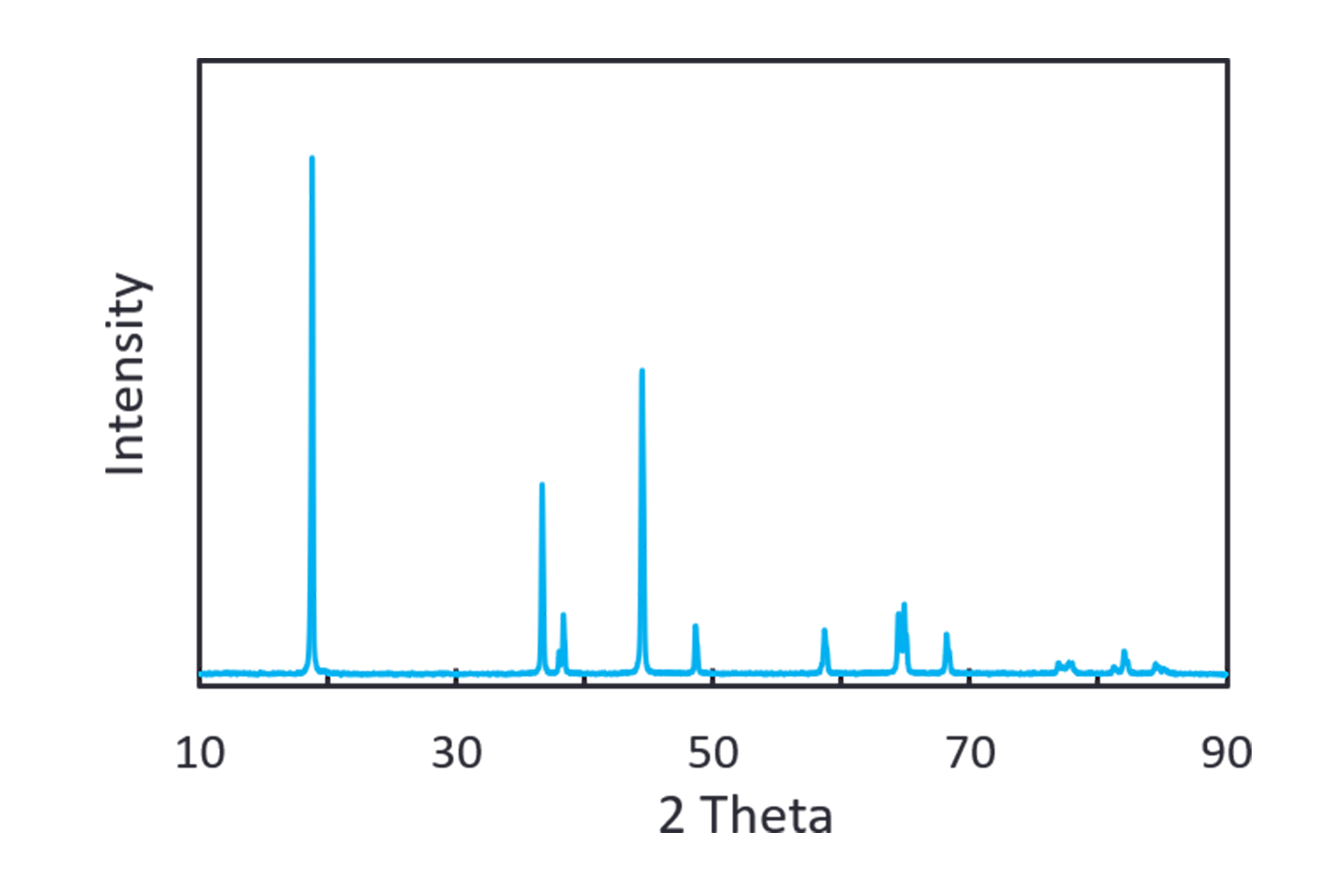


**Figure S1.** XRD patterns of commercial NCM811 polycrystalline particles


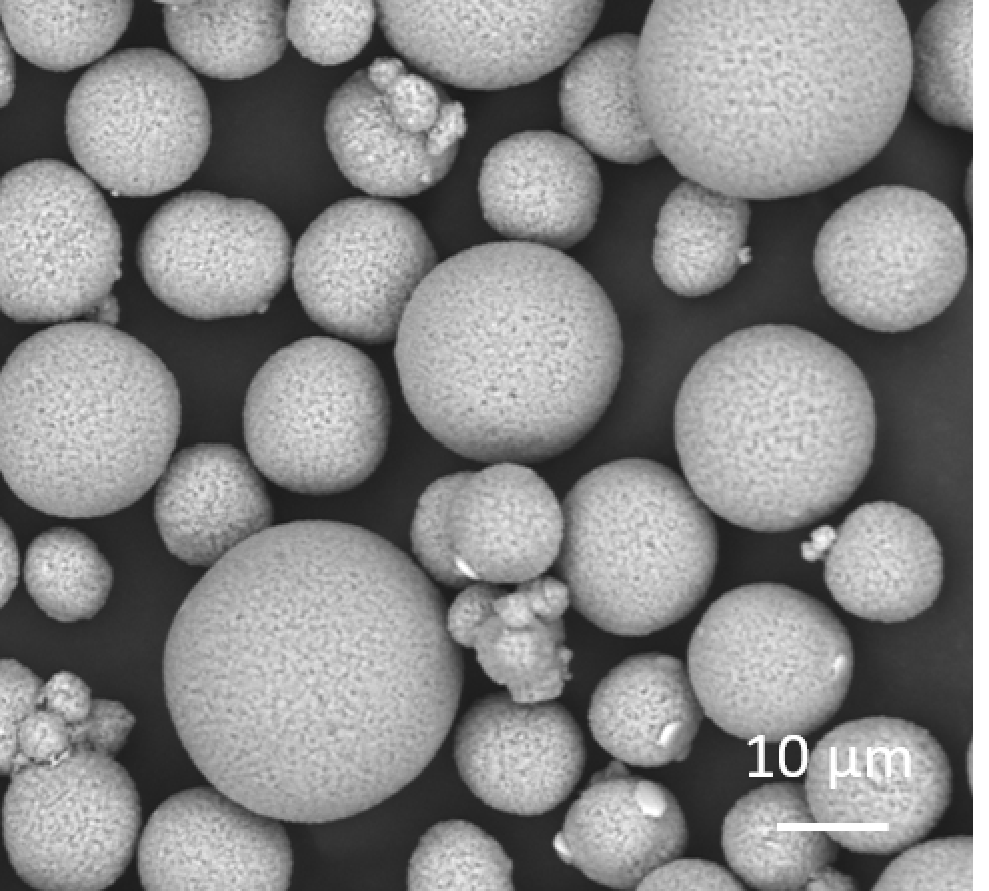


**Figure S2.** SEM image of commercial NCM811 polycrystalline particles.


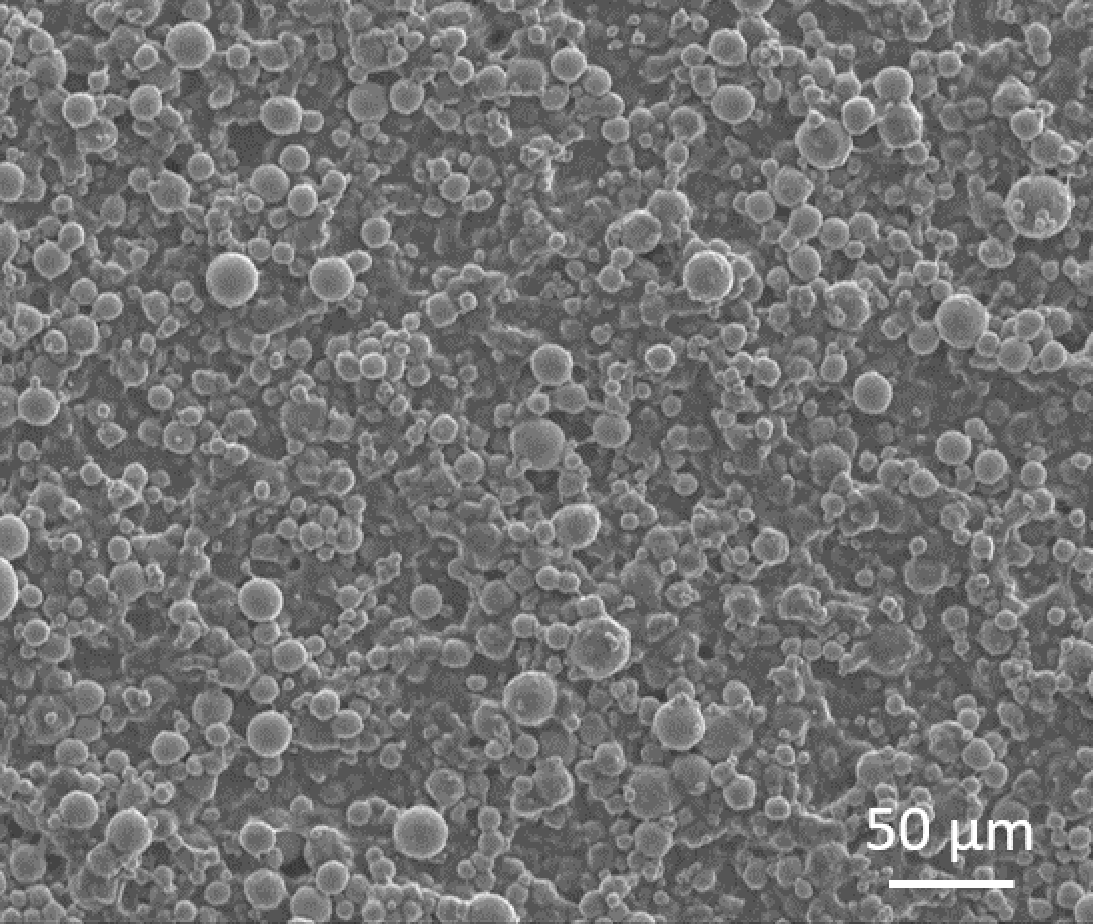


**Figure S3.** SEM image of the as-prepared solid-state NCM811 electrode.


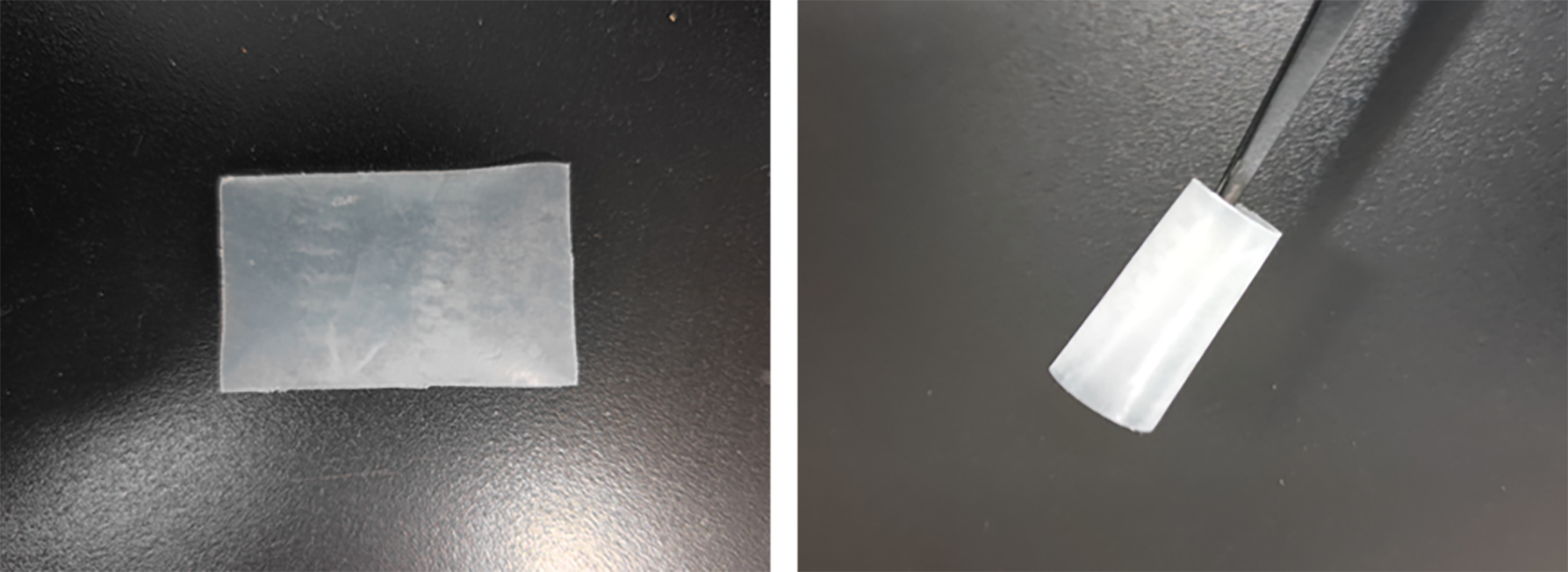


**Figure S4.** The photographs of the as-prepared flexible solid polymer electrolyte films.


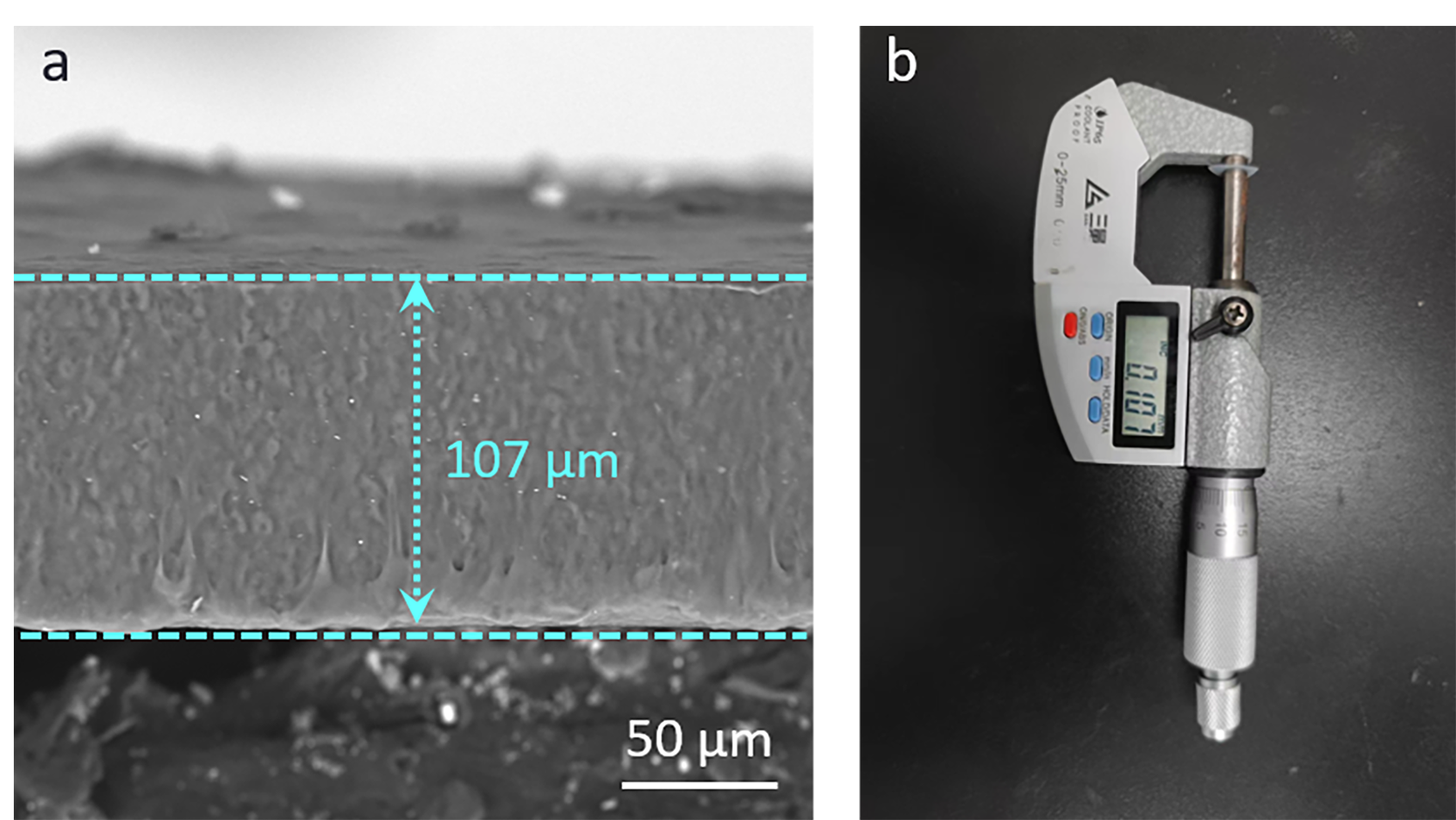


**Figure S5.** (a) The SEM image of the cross section of as-prepared solid polymer electrolyte films. (b) The thickness test of as-prepared solid polymer electrolyte films.


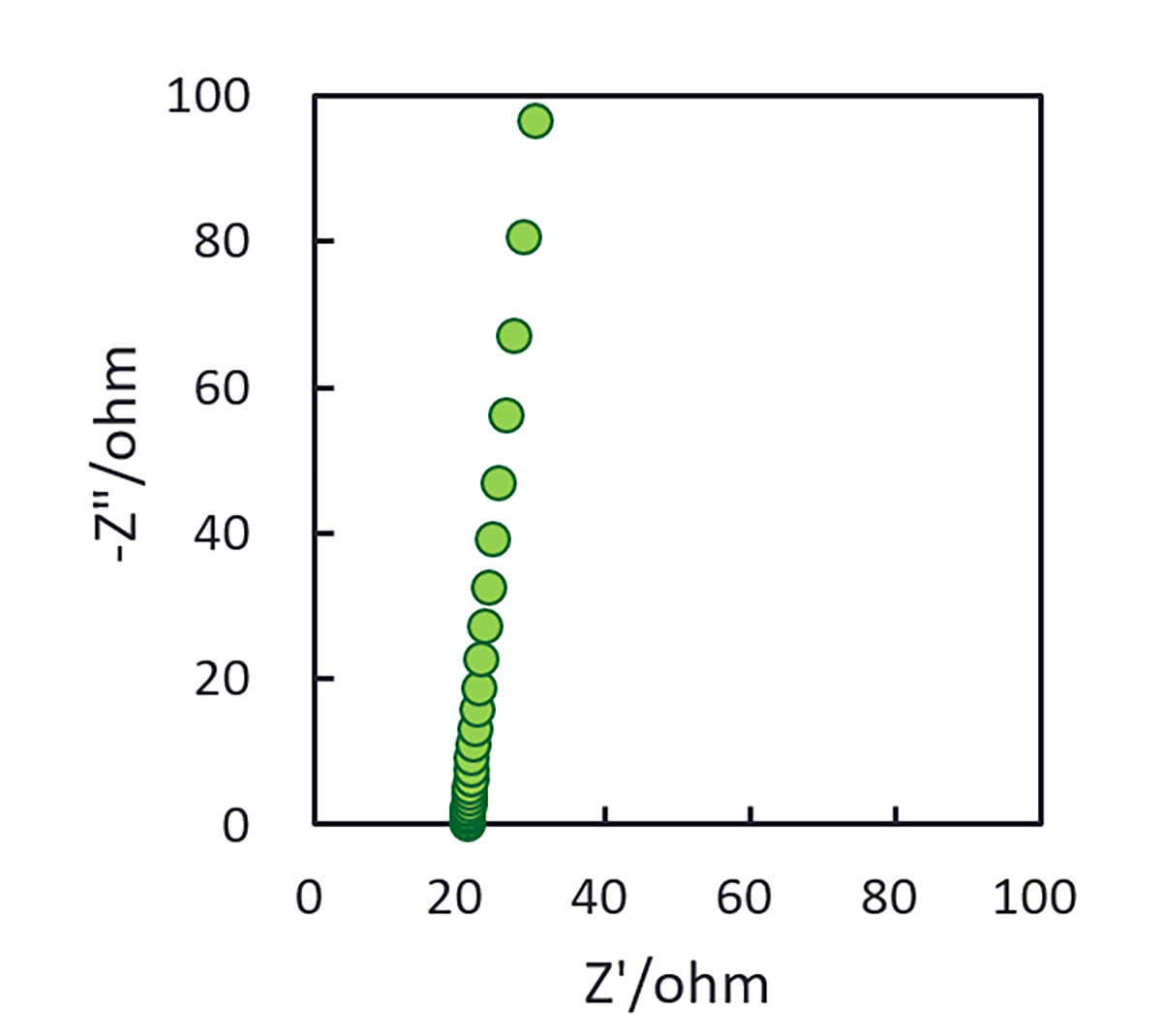


**Figure S6.** Electrochemical impedance spectroscopy of the Li|SSE|Li cells.


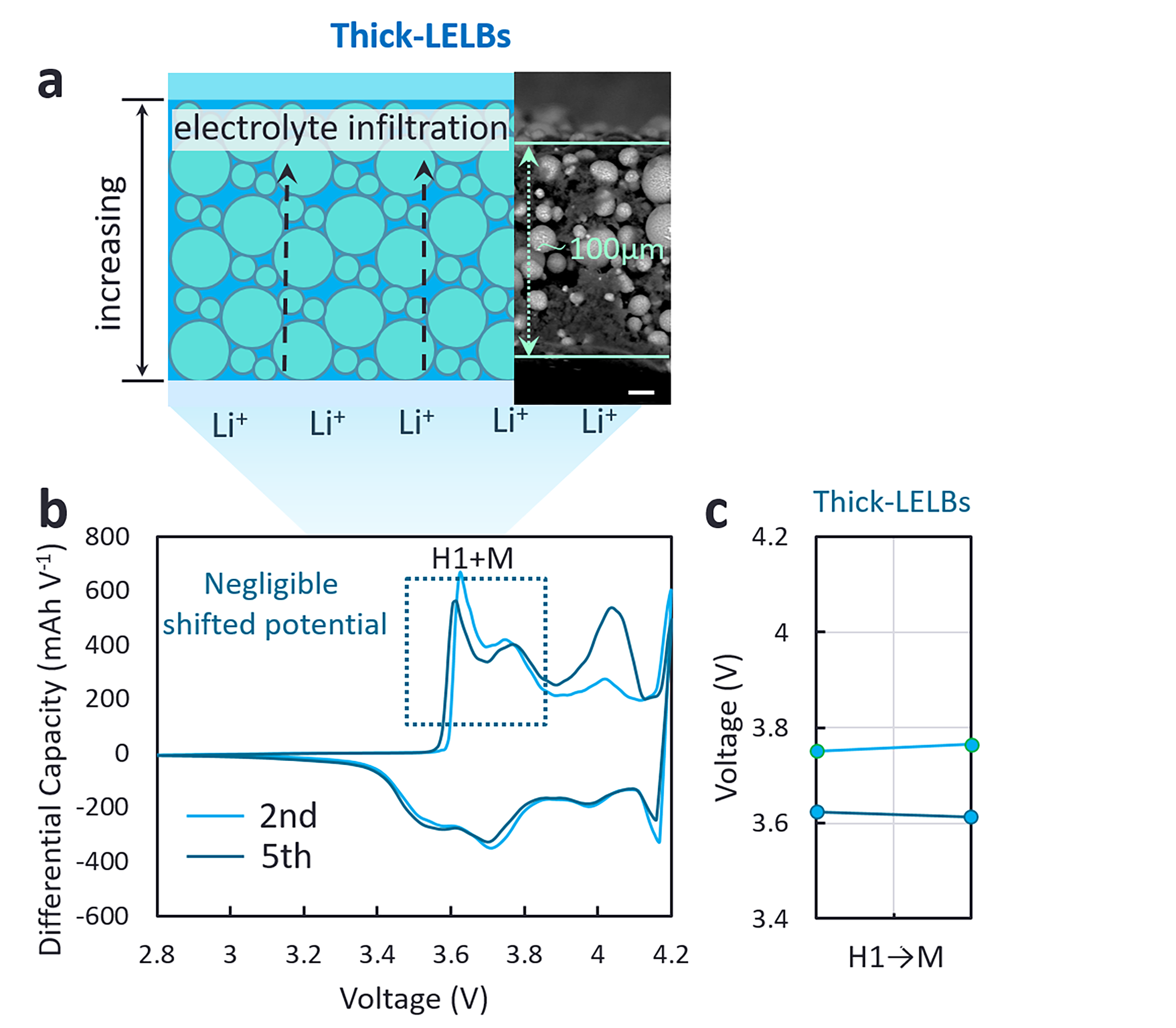


**Figure S7.** (a) Schematic diagram of thick-LELBs and thick-ASSLB. (b) dQ/dV profiles at 2nd cycle and 5th cycle for thick-LELBs. (c) The potential of H1 to M at 2nd cycle and 5th cycle in thick-LELBs.


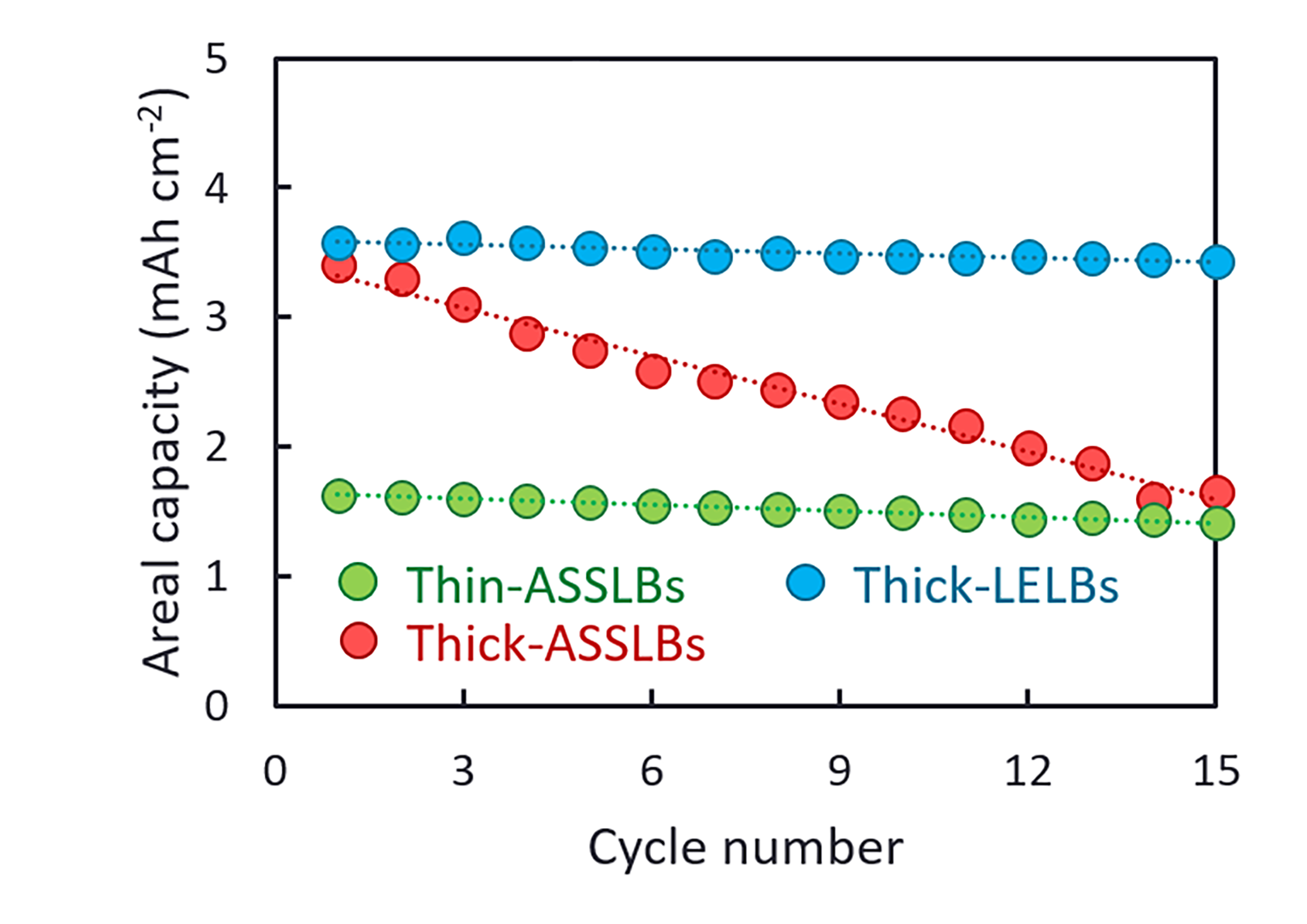


**Figure S8.** Comparison of the areal capacity of thin-ASSLBs, thick-ASSLBs, and thick-LELBs under 0.1C.


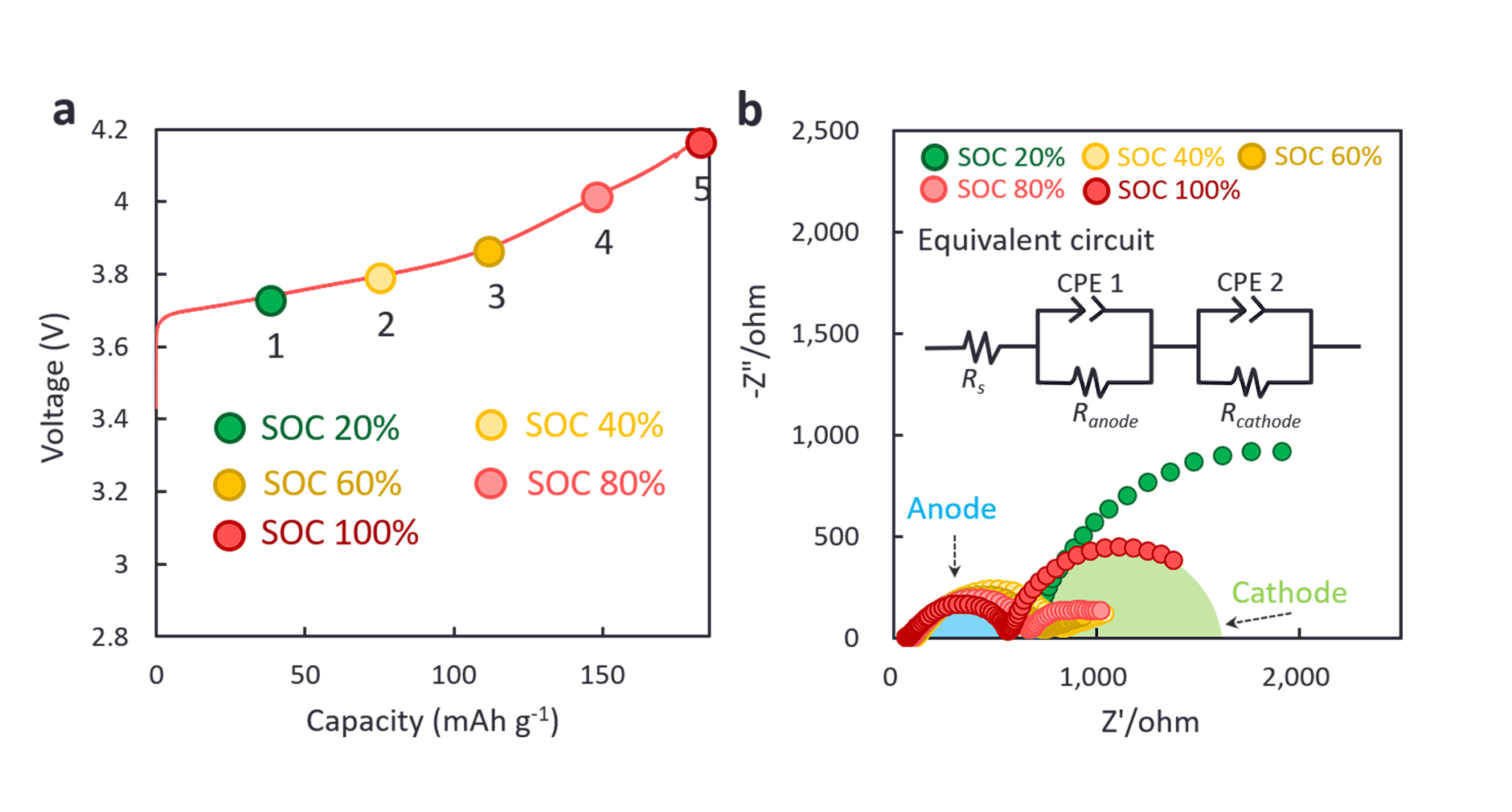


**Figure S9.** In situ EIS of the thick-ASSLBs during the initial charging process. (a). Selected points with equal intervals in the charging process. (b). Evolution of the EIS results in the charging process, equivalent circuit, and illustration of the EIS model for the thick-ASSLBs, corresponding to the selected points in Figure S6a.


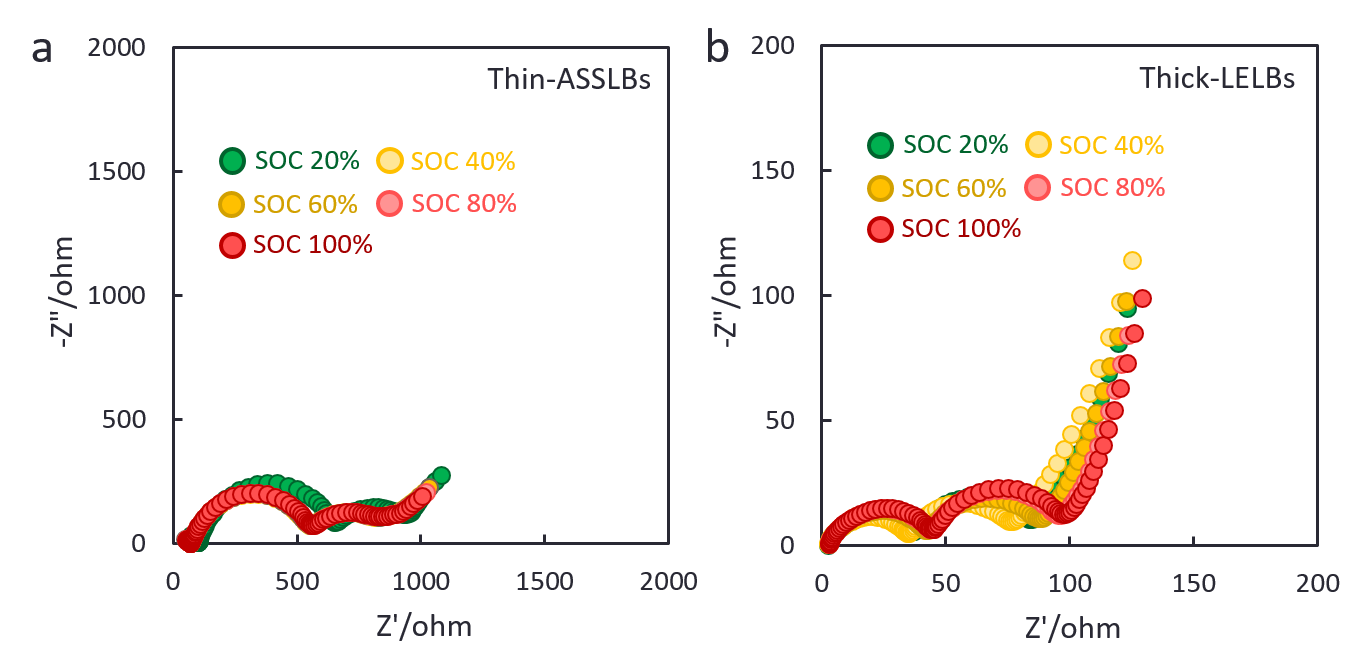


**Figure S10.** In situ EIS results of different SOC of (a) the thin-ASSLBs and (b) thick-LELBs during the initial charging process.


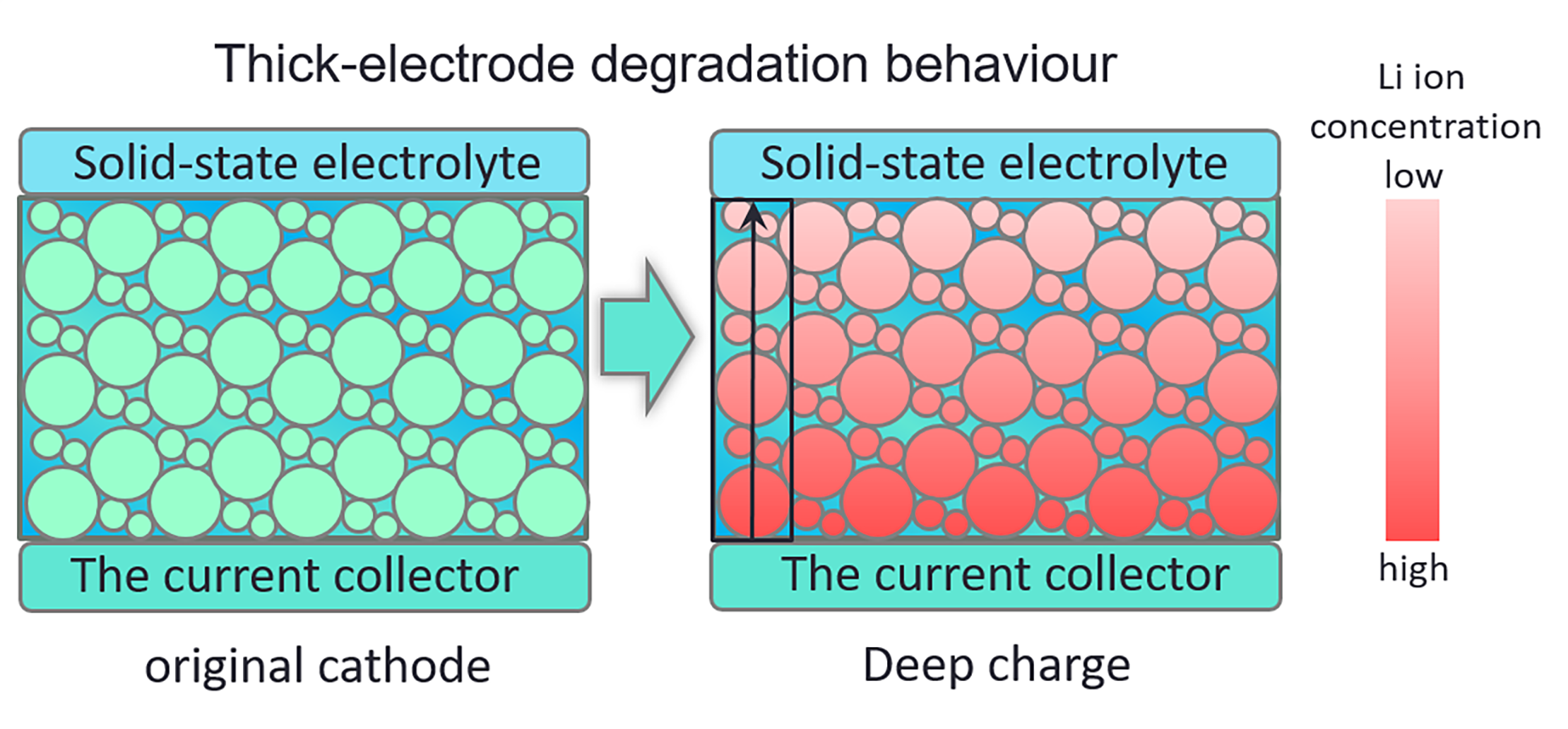


**Figure S11.** Schematic diagram of electrode degradation behavior in thick-ASSLBs.


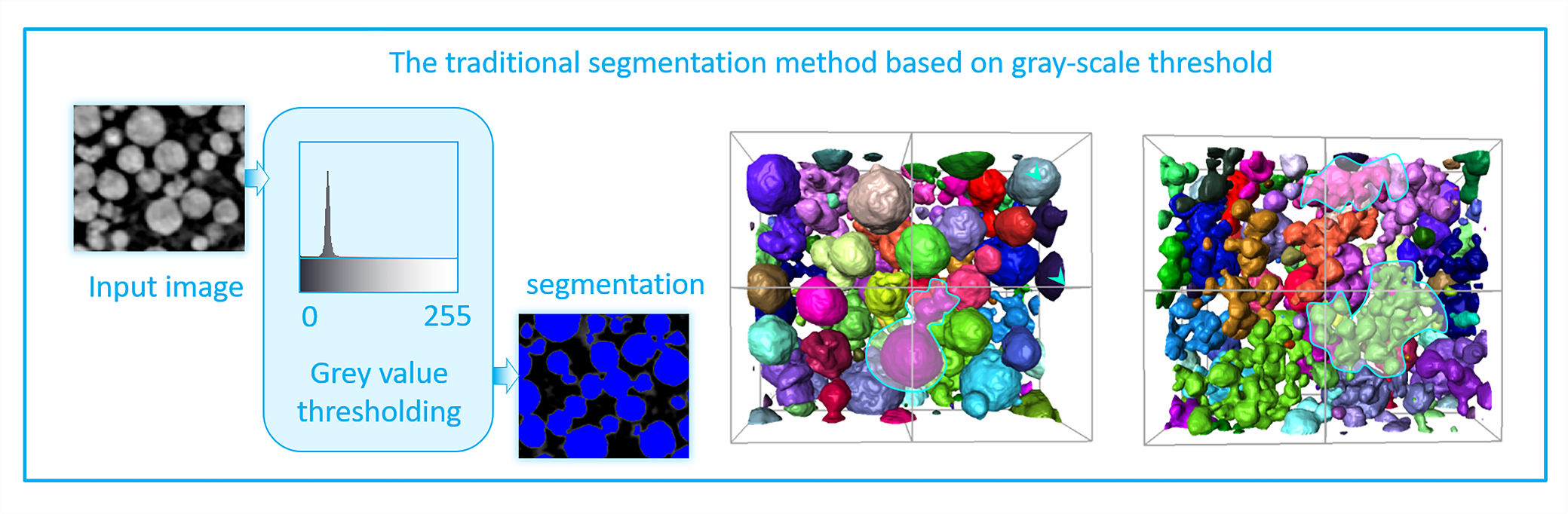


**Figure S12.** Schematic diagram of the traditional segmentation method based on gray-scale threshold.


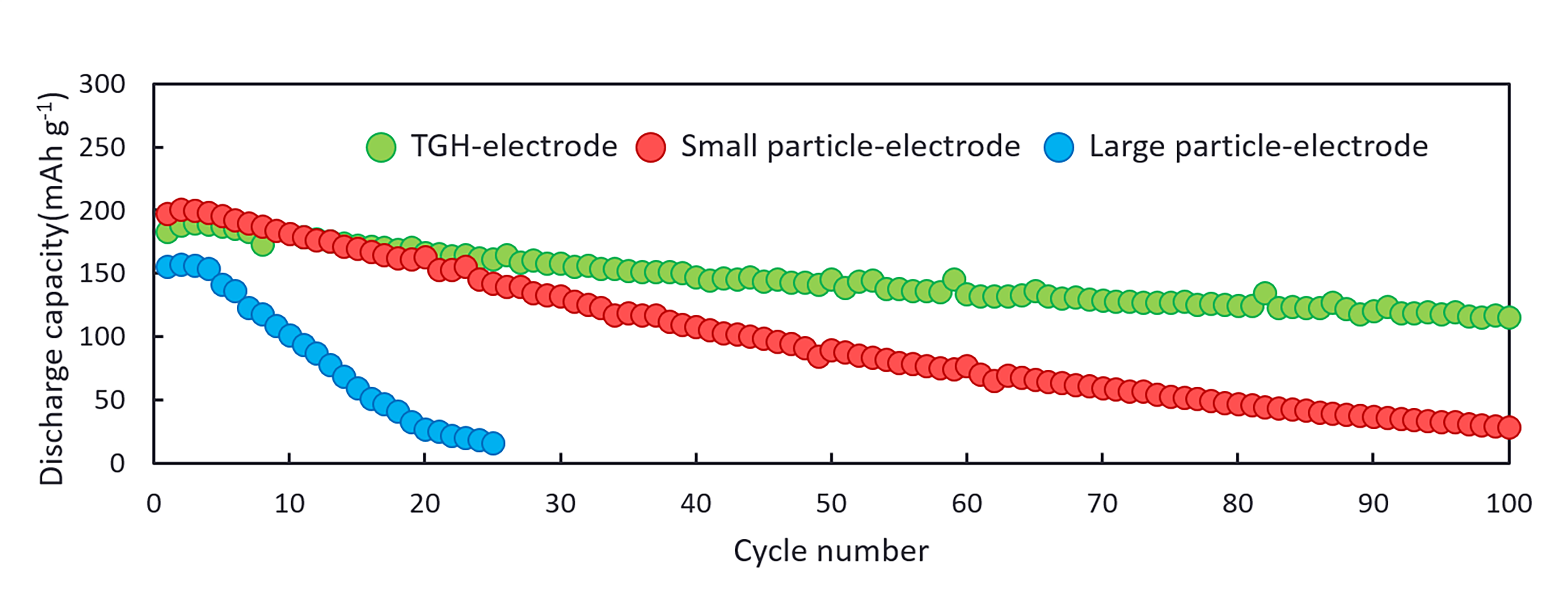


**Figure S13.** Cycling stability comparison among TGH-electrode, small particle-electrode, and large particle-electrode under 0.1C.


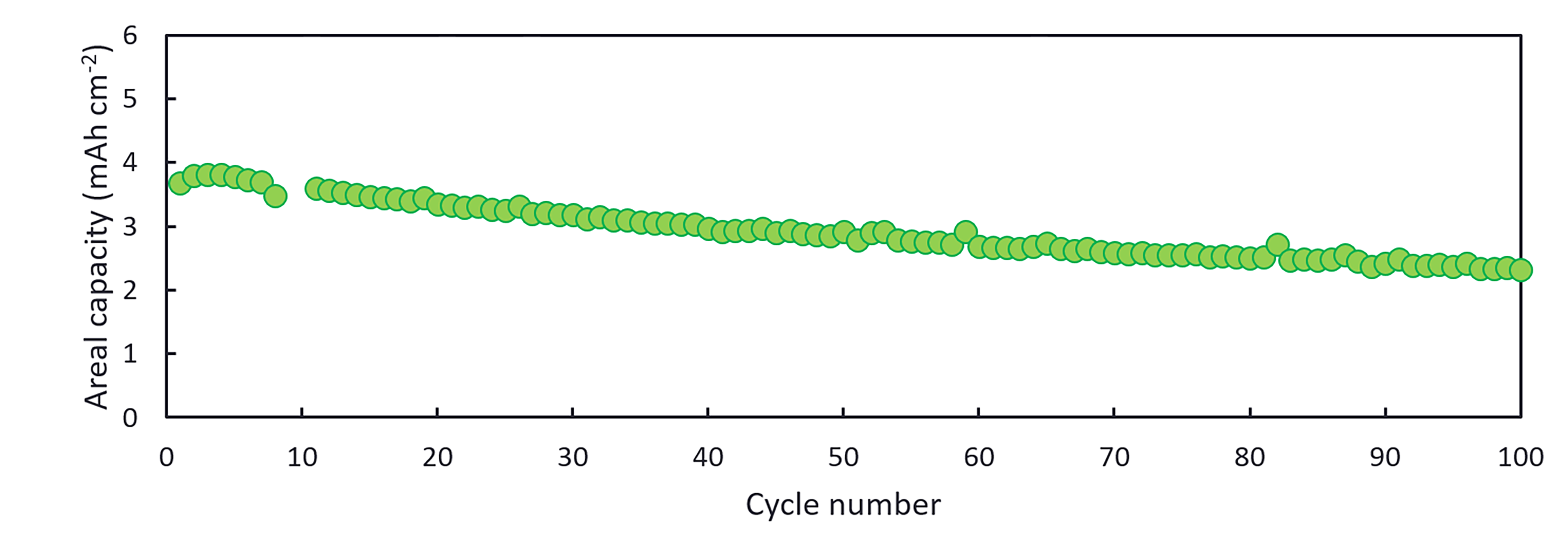


**Figure S14.** The areal capacity of TGH-electrode under 0.1C.


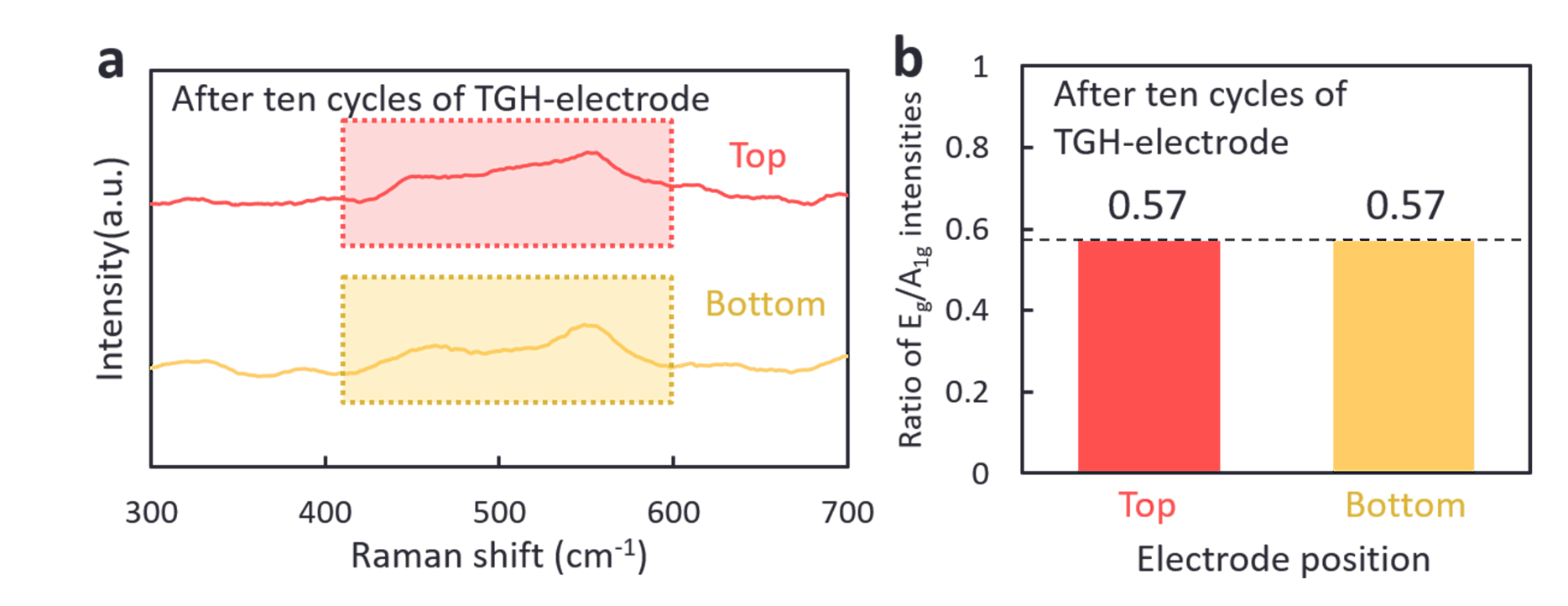


**Figure S15.** Raman spectra of cycled TGH-electrode. (a) Raman spectra of the NCM particles at the top and bottom of cycled TGH-electrode. (b) Ratio of E_g_/A_1g_ intensities at the top and bottom of TGH-electrode.


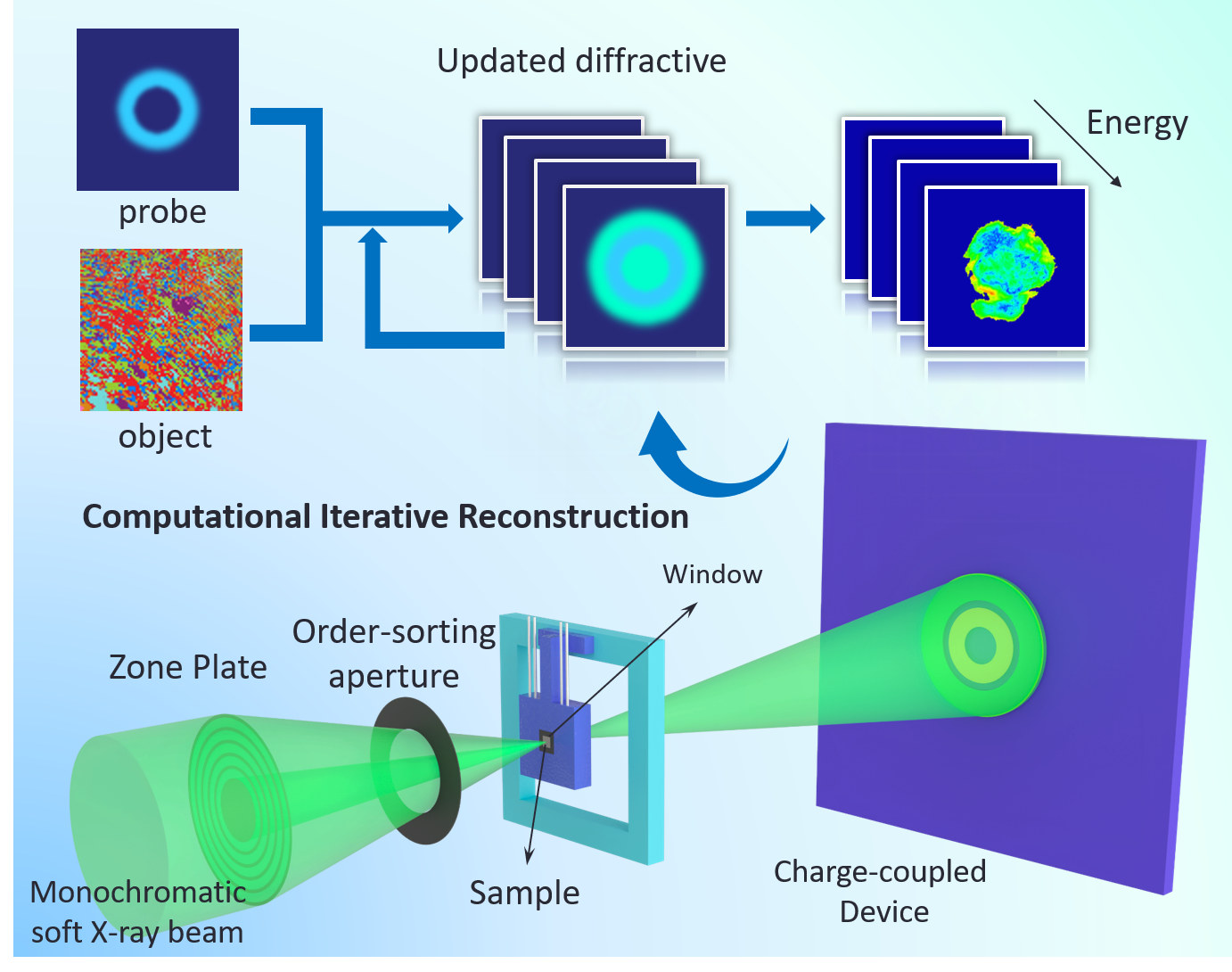


**Figure S16.** Schematic diagram of the STXM-based soft X-ray Ptychography.


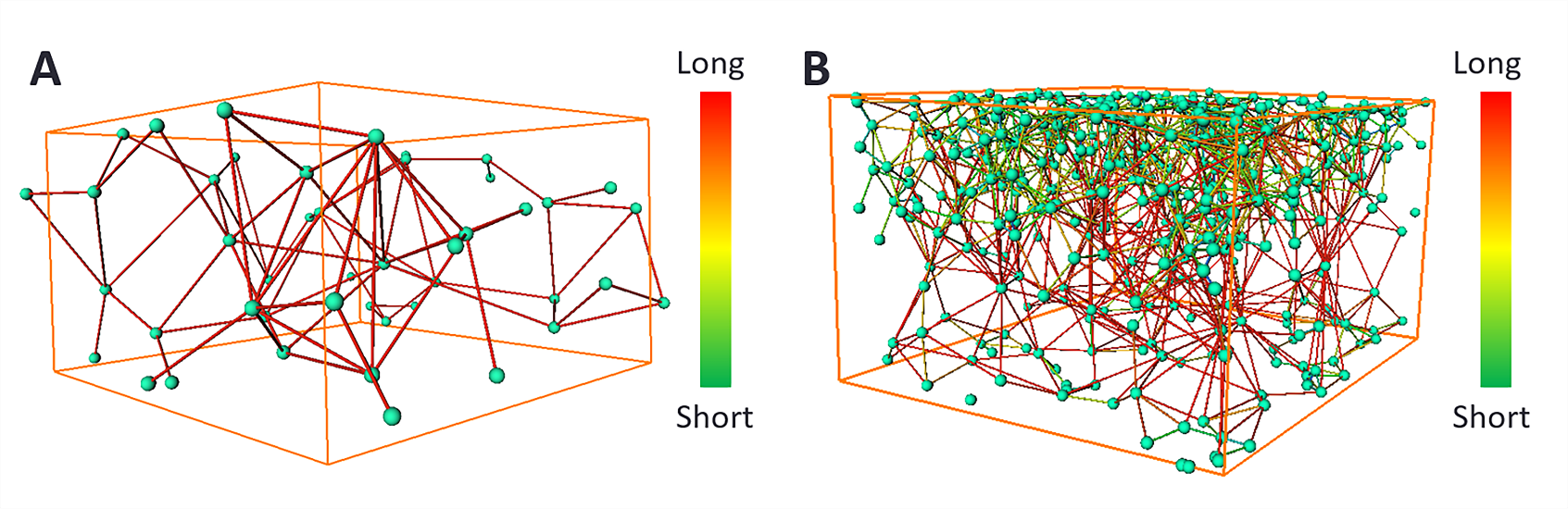


**Figure S17.** The connectivity network model based on synchrotron x-ray tomography. The connectivity network models of (a) the regular cathodes and (b) TGH-electrode, the intensity bar represents the transport distance in the connectivity network.


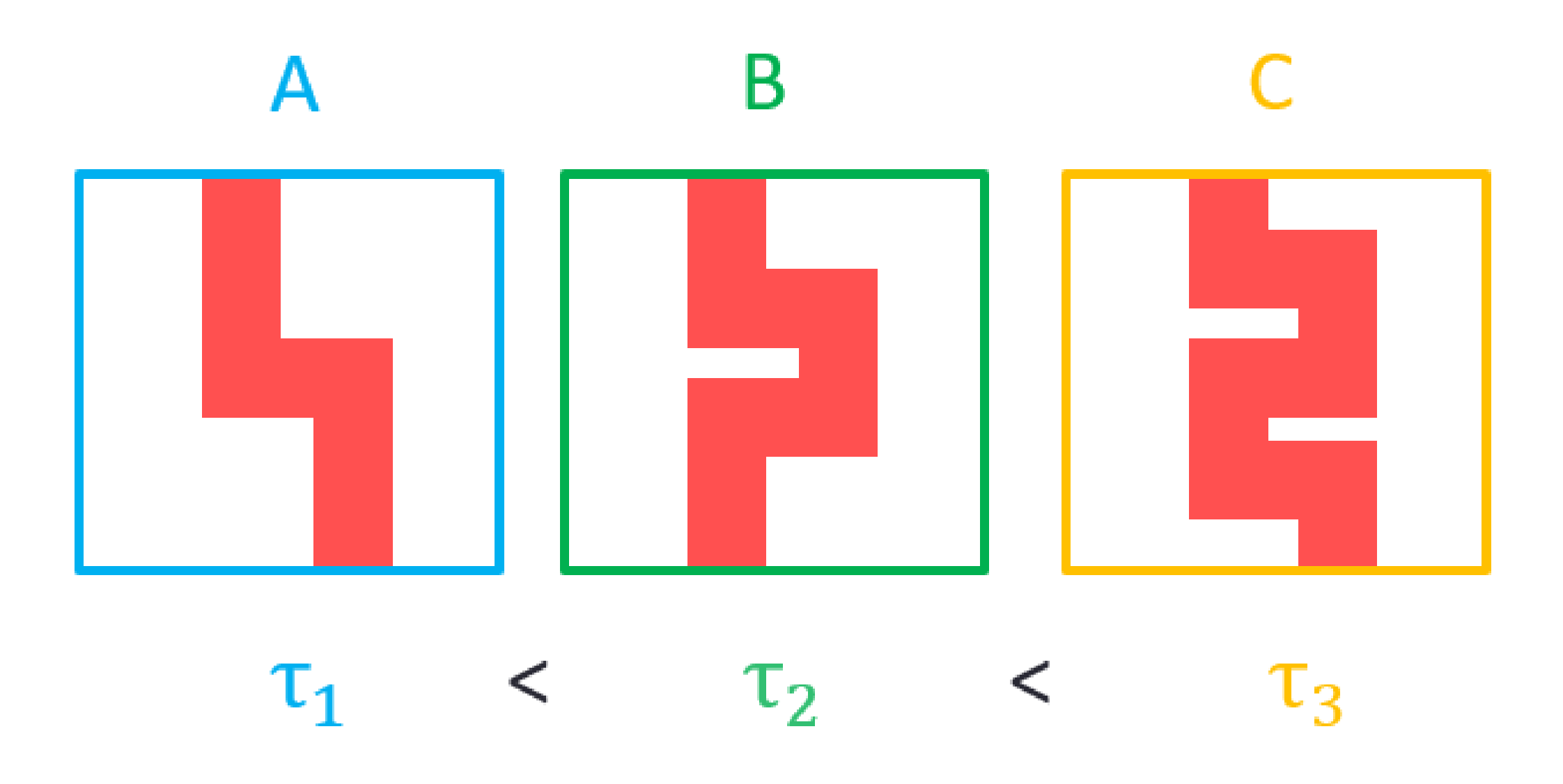


**Figure S18.** An explanation of the different types of pores that can exist in the porous electrode and their influence on the tortuosity factor.


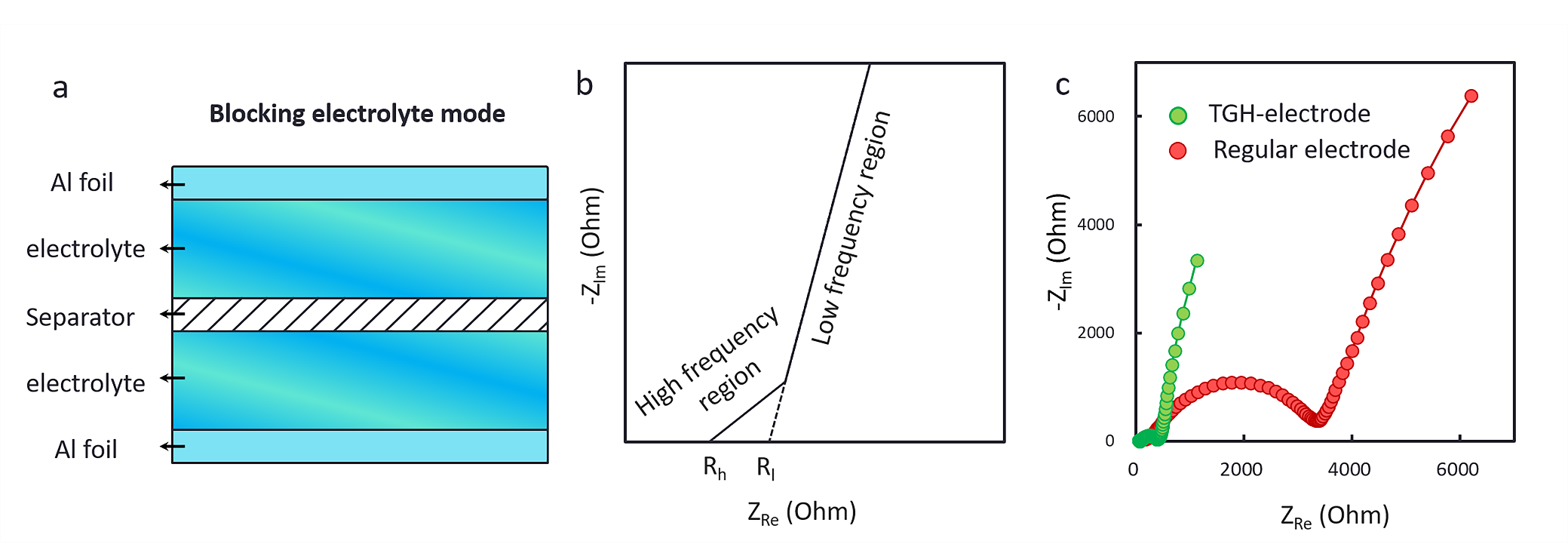


**Figure S19.** The blocking-electrolyte impedance method for electrode tortuosity. (a) The schematic of blocking-electrolyte cell. (b) The schematic of Nyquist impedance plot with the position of R_h_ and R_l_. (c) The EIS results of TGH-electrode and Regular-electrode.


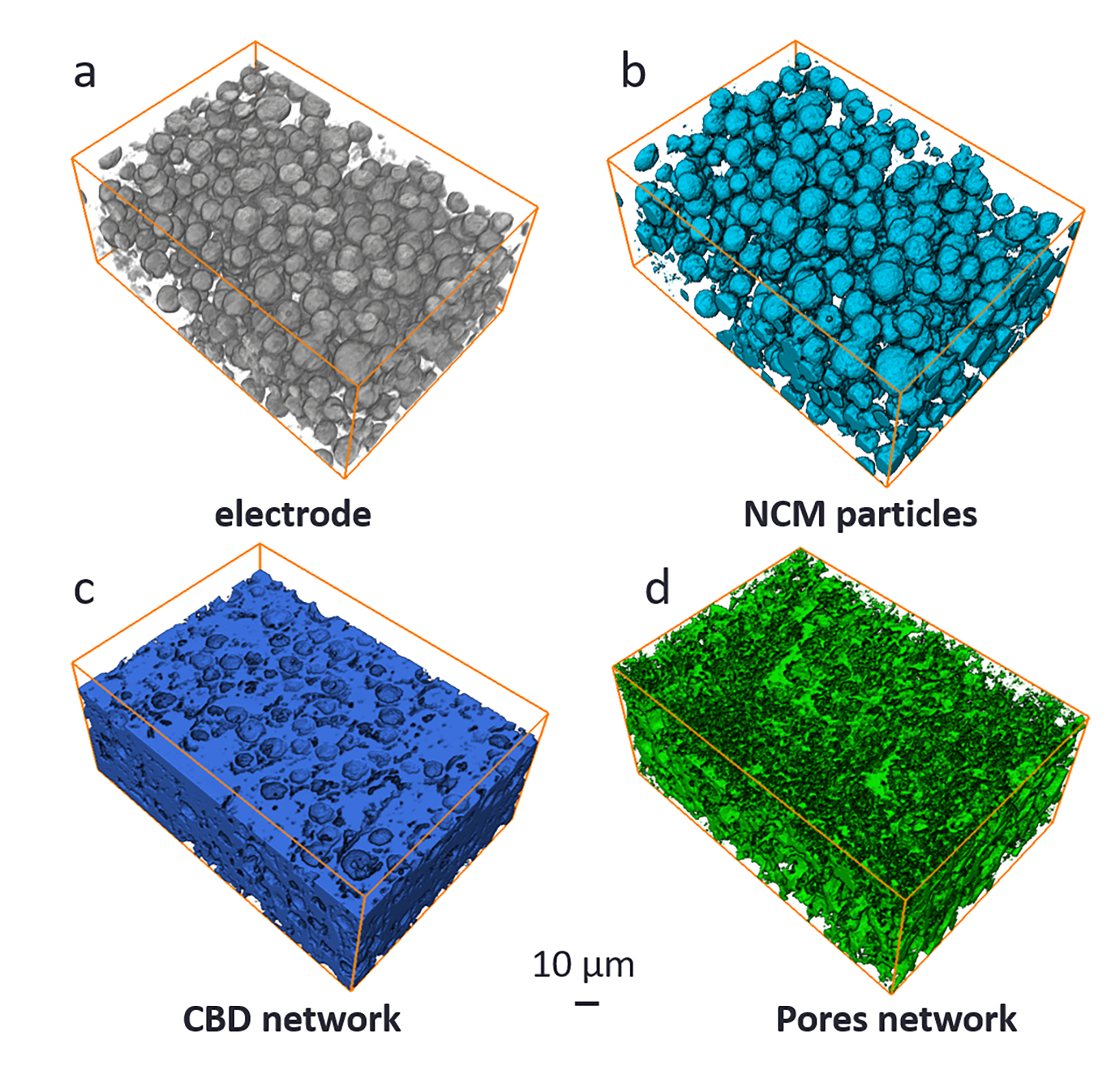


**Figure S20.** (a) Synchrotron x-ray tomography reconstruction with volume rendering shows 3D microstructure of the regular electrode. (b) The particle phase, (c) CBD network (crop a part for clarity view) and (d) pore network were identified by machine learning method.


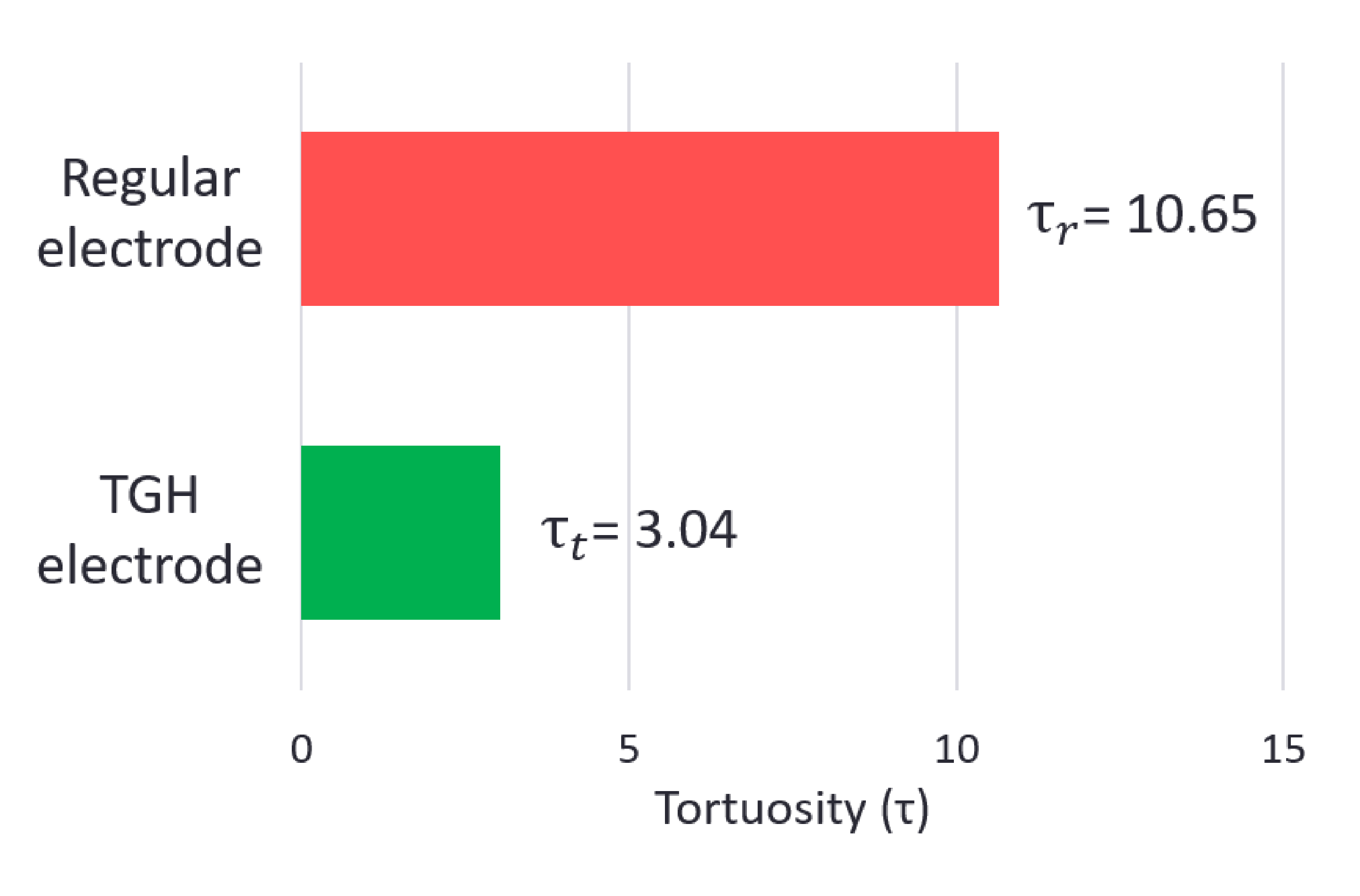


**Figure S21.** The tortuosity of regular electrode and TGH-electrode.


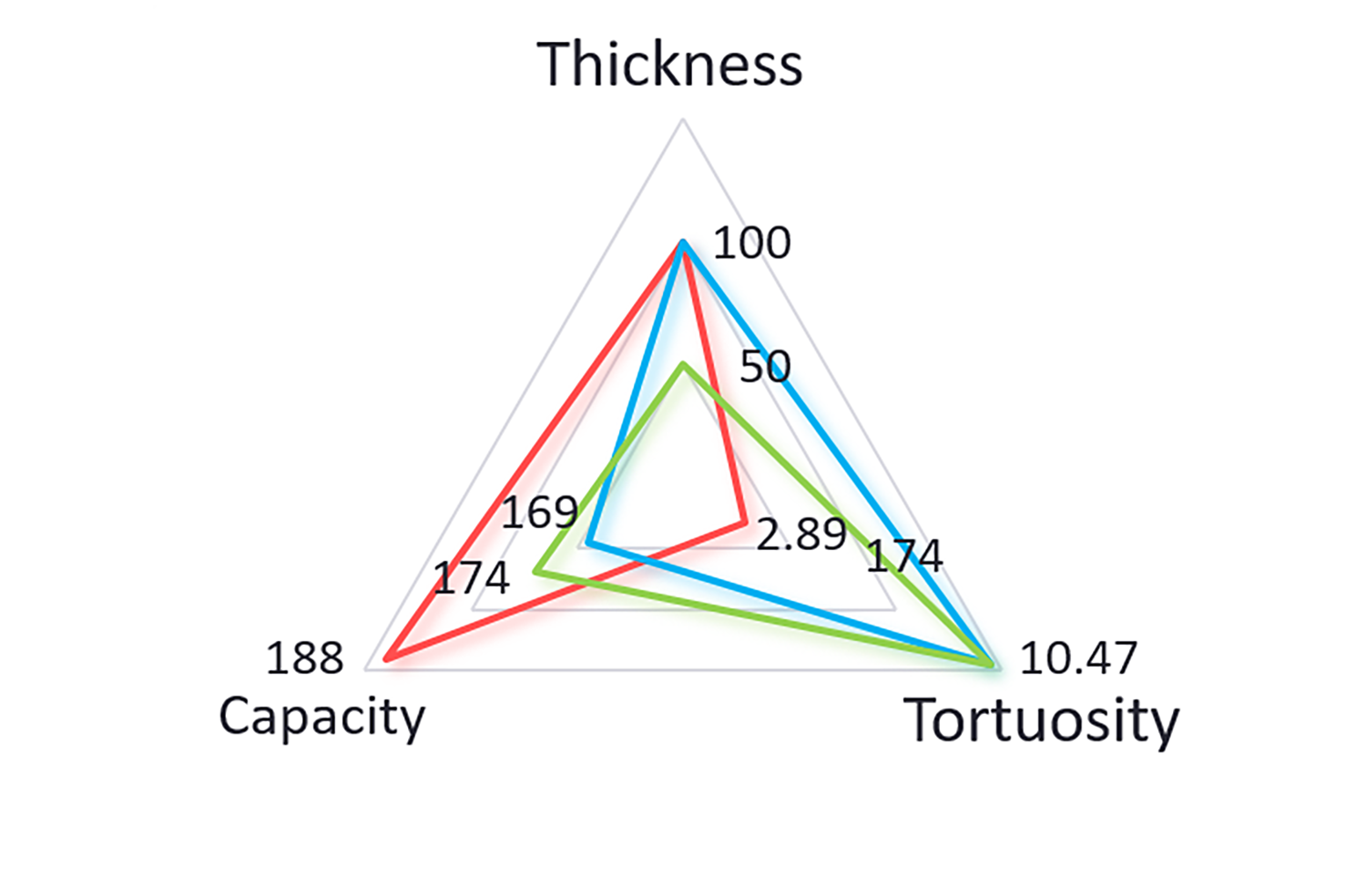


**Figure S22.** The statistical analysis of the capacity, thickness, and tortuosity.

**REFERENCES**

1. Chen H, Zhou G and Boyle D et al. Electrode design with integration of high tortuosity and sulfur-philicity for high-performance lithium-sulfur battery. Matter 2020; 2: 1605-20.

2. Wang X, Xiao R and Xiang Y et al. Density functional investigation on cathode/electrolyte interface in solid-state lithium batteries. J Electrochem 2017; 23: 381-390.
